# Mechanical compression creates a quiescent muscle stem cell niche

**DOI:** 10.1038/s42003-023-04411-2

**Published:** 2023-01-13

**Authors:** Jiaxiang Tao, Mohammad Ikbal Choudhury, Debonil Maity, Taeki Kim, Sean X. Sun, Chen-Ming Fan

**Affiliations:** 1 Embryology Department, Carnegie Institution for Science, 3520 San Martin Drive, Baltimore, MD 21218 USA; 2 Department of Mechanical Engineering, Johns Hopkins University, Baltimore, MD 21218 USA; 3 Institution for NanoBioTechnology, Johns Hopkins University, Baltimore, MD 21218 USA; 4 Department of Civil & Systems Engineering, Johns Hopkins University, Baltimore, MD 21218 USA; 5 Center for Cell Dynamics (CCD), Johns Hopkins School of Medicine, Baltimore, MD 21205 USA; 6 Department of Biology, Johns Hopkins University, Baltimore, MD 21218 USA

**Keywords:** Biophysics, Stem cells

## Abstract

Tissue stem cell niches are regulated by their mechanical environment, notably the extracellular matrix (ECM). Skeletal muscles consist of bundled myofibers for force transmission. Within this macroscopic architecture, quiescent Pax7-expressing (Pax7^+^) muscle stem cells (MuSCs) are compressed between ECM basally and myofiber apically. Muscle injury causes MuSCs to lose apical compression from the myofiber and re-enter the cell cycle for regeneration. While ECM elasticities have been shown to affect MuSC’s renewal, the significance of apical compression remains unknown. To investigate the role of apical compression, we simulate the MuSCs’ in vivo mechanical environment by applying physical compression to MuSCs’ apical surface. We demonstrate that compression drives activated MuSCs back to a quiescent stem cell state, regardless of basal elasticities and chemistries. By mathematical modeling and cell tension manipulation, we conclude that low overall tension combined with high axial tension generated by compression leads to MuSCs’ stemness and quiescence. Unexpectedly, we discovered that apical compression results in up-regulation of Notch downstream genes, accompanied by the increased levels of nuclear Notch1&3 in a Delta ligand (Dll) and ADAM10/17 independent manner. Our results fill a knowledge gap on the role of apical compression for MuSC fate and have implications to stem cells in other tissues.

## Abstract (web summary)

Mechanical compression drives activated muscle stem cells (MuSCs) into a quiescent stem cell state providing insight into MuSC activity during injury-regeneration cycles.

## Introduction

Skeletal muscles consist of post-mitotic syncytial myofibrils that generate contractile forces for body movement^1^. They have a tremendous ability to regenerate after injury mainly owing to the resident Pax7-expressing (Pax7^+^) muscle stem cells (MuSCs)^2–5^, also known as satellite cells^1,2^. Within the uninjured muscle, MuSCs are mostly quiescent and sandwiched between the basement membrane (i.e., ECM) and myofiber. Upon muscle damage, MuSCs lose apical contact with the myofiber but retain contact with the ECM of the dead myofiber (i.e., the ghost fiber^6^). They then re-enter the cell cycle, become myogenically committed, and later differentiate and fuse to form new myofibers^7^. Some proliferative MuSCs self-renew and return to quiescence to maintain the stem cell pool as the regenerative cycle completes^8^. Molecular and genetic studies have uncovered genes and pathways regulating progressive states of MuSCs during the regenerative cycle^9^. On the other hand, mechanical stimuli have also been shown to regulate MuSCs’ fate. Mechanisms underlying how forces can trigger relevant endogenous signaling pathways are currently limited to cell–ECM interaction. For example, laminin-coated hydrogels with a stiffness of 12 kPa, close to the physiological elasticity^10^, provide an optimal condition for MuSC self-renewal in culture^10–13^; whereas culturing them on 2 kPa collagen I fibrils in synthetic media has been shown to prevent their activation^14^. Here, we address the role of understudied apical compression for the quiescence state of MuSCs.

## Results

### Quiescent MuSCs change morphology during the transition to activated state

From the intravital imaging data of yellow fluorescent protein (YFP)-marked Pax7^+^ MuSCs^6^, MuSCs display a flat and elongated shape in uninjured muscle: cell dimension perpendicular to myofiber (height) is ~4 μm, and cell dimension parallel to myofiber (length) is ~17 μm. At 1-day post-injury (dpi), they are ~5.5 μm in cell height and ~6 μm in cell length (Supplementary Fig. 1a–c). Compression release from the myofiber after injury likely contributes to such morphological changes. At 3 dpi, many YFP-marked cells are proliferating and/or migrating, and their cell height and length are both ~10 μm (with dynamically changing lengths), which are both larger compared to their uninjured counterparts (Supplementary Fig. 1d, e). We were particularly intrigued by the cell shape change at 1 dpi before a detectable MuSC proliferation at 2 dpi^6^ and that MuSCs on uninjured myofibers immediately adjacent to an injury site does not appear activated^6^. We hypothesize that compression force exerted on MuSCs by intact myofibers keeps them quiescent and tested our hypothesis using cultured MuSCs as described below.

### Apical compression increases quiescent MuSCs

**Fig. 1:**
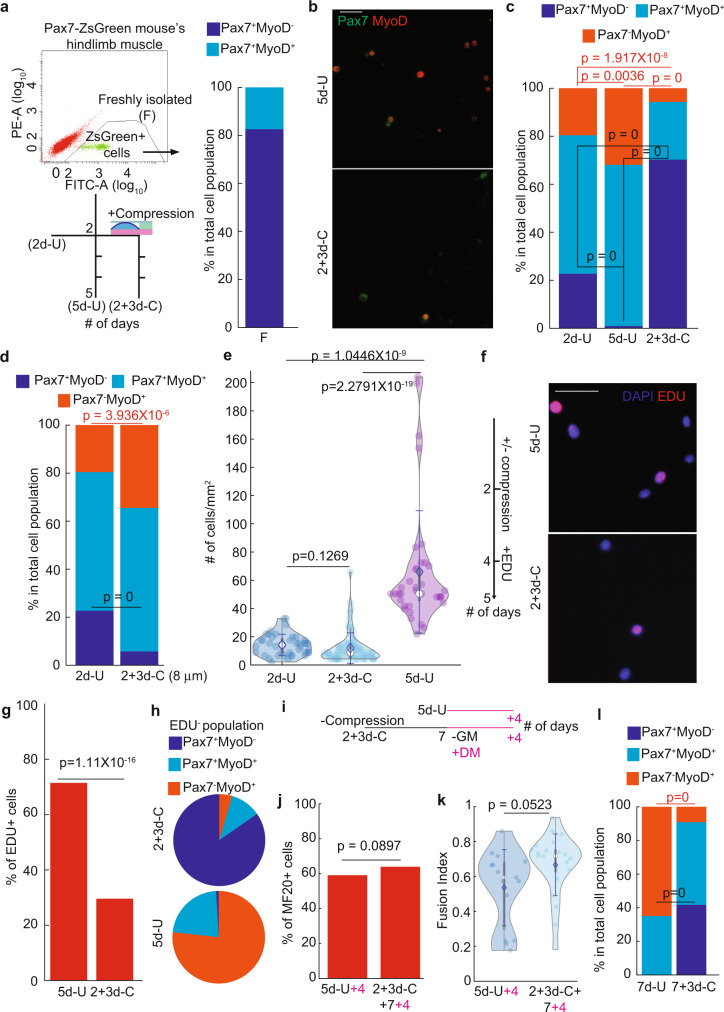
Manipulating cell fate using mechanical compression. **a** Experimental and FACS gating schemes. Cell fate evaluation of freshly isolated (F) cells by Cytospin (*n* = 3; 741 cells). **b** Examples of Pax7 and MyoD expression of 5d-U and 2+3d-C cells (Scale bar, 25 μm; Split channel images with DAPI in Supplementary Fig. 4b). **c** Cell fate evaluation of 2d-U, 5d-U, and 2+3d-C cells (*n* = 4 for 2d-U, of total 260 cells; *n* = 5 for 5d-U, of total 3752 cells; *n* = 13 for 2+3d-C, of total 420 cells). **d** Cell fate comparison between 2d-U and 2+3d-C (8 μm) cells. (2d-U data is the same with (**c**); *n* = 6 for 2+3d-C (8 μm), of total 1032 cells). **e** Cell densities comparison between 2d-U, 5d-U, and 2+3d-C cells (same data sets presented in (**c**): 40 fields of view for 2d-U, 73 fields of view for 2+3d-C, and 37 fields of view for 5d-U). **f** EDU incorporation setup and example images of 5d-U and 2+3d-C cells (scale bar, 25 μm; Split channel images with DAPI in Supplementary Fig. 4f). **g** and **h** Fraction of EDU^+^ cells of 5d-U and 2+3d-C cells (**g**), and cell fate component of EDU^−^ population of 5d-U and 2+3d-C cells (**h**) (*n* = 6 for 5d-U, of total 3752 cells; *n* = 8 for 2+3d-C, of total 180 cells). **i** Experimental setup to test the redifferentiation capacity of compressed cells. GM growth medium, DM differentiation medium. **j** and **k** Redifferentiation capacity of compressed cells compared to that of 5d-U cells: fraction of MF20^+^ population (**j**), and fusion index (**k**) (*n* = 4 for 2+3d-C+4+7; of total 810 cells; *n* = 3 for 5d-U + 4, of total 450 cells.). **l** Cell fate comparison between 7d-U and 7+3d-C cells (*n* = 3 for 7d-U, of total 1918 cells; *n* = 3 for 7+3d-C, of total 602 cells). Data in **c**, **d**, **g**, **j**, and **l** was presented with an overall fraction counting every cell from all experimental repeats. *p-*value was assessed by a two-tailed Cochran–Mantel–Haenszel test using MATLAB. **e** and **k** data was presented with mean $\pm$ standard deviation (s.d). *p-*value was assessed with the student’s two-tail *t*-test (**k**) and Kruskal–Wallis tests (**e**) using MATLAB. The comparison was considered significant if $p\le 0.05$. Additional representative images and split channel images with DAPI for panels **b** and **f** are shown in Supplementary Figs. 4 and  5.

To investigate the link between cellular morphology/mechanical tension and MuSC state, we established a system to simulate the apical loading on quiescent MuSCs. We microfabricated a polydimethylsiloxane (PDMS) device consisting of a thin top film with vertical pillars of ~4 μm in height underneath (Supplementary Fig. 2a–c; see the “Methods” section)^15,16^. MuSCs cultured under this device was presumably limited to a height of 4 μm, mimicking their intact physical niche in vivo. Pax7^+^ MuSCs were isolated from transgenic Pax7-ZsGreen mice^17^ by fluorescence-activated cell sorting (FACS, Fig. 1a). They were seeded on Matrigel/fibronectin-coated plastic dish and cultured for 2 days in growth media (see the “Methods” section). At this time, the cell height was measured at ~8.7 μm using confocal imaging. After adding the compression device, we confirmed that cells were at the expected height of ~4 μm (Supplementary Fig. 3a, b).

We evaluated the role of compression on MuSCs’ fate, by comparing Pax7 and MyoD expression between freshly isolated, compressed, and uncompressed MuSCs: Pax7^+^MyoD^−^ for stem cells, Pax7^+^MyoD^+^ for progenitors, and Pax7^−^MyoD^+^ for differentiation committed cells^18^. Freshly isolated MuSCs (F) were all Pax7^+^, with some also expressing MyoD (Fig. 1a; Supplementary Fig. 4a). After the initial 2 days of culture without compression (2d-U), most cells expressed MyoD (~70%) with some Pax7^−^ cells, indicative of MuSC activation and differentiation. MuSCs were then either left uncompressed for an additional 3 days (5d-U) or subjected to compression for the same period (2 + 3d-C). The Pax7^+^MyoD^−^ cells were almost depleted in the 5d-U. By contrast, 2 + 3d-C MuSCs contained a significantly larger fraction of Pax7^+^MyoD^−^ cells and smaller fractions of both Pax7^+^MyoD^+^ and Pax7^−^MyoD^+^ cells compared to both the 5d-U and 2d-U conditions (Fig. 1b, c; Supplementary Fig. 4b for split-channel images with DAPI and representative images for F and 2d-U cells). To address that cell fate was affected by compression rather than cell-PDMS contact, we deployed a compression device with 8 μm-high pillars for minimal compression (~10% compression, 2 + 3d-C (8 μm)). A similar cell fate distribution was found between 2 + 3d-C (8 μm) and 5d-U cells, supporting that 4 μm height with ~55% compression is needed to cause cell fate change (Fig. 1d; Supplementary Fig. 4c). We also found a similar trend of cell fate changes among MuSCs that were 1-day uncompressed (1d-U), 4-day uncompressed (4d-U), and 3-day under compression after initial 1-day uncompressed culture condition (1 + 3d-C), supporting that compression is able to preserve Pax7^+^MyoD^−^ stem cells (Supplementary Fig. 4d, e). We noted that 2 + 3d-C cells had a similar but much lower cell density compared to 2d-U and 5d-U cells, respectively (Fig. 1e). We performed an EdU-incorporation assay and determined that 2 + 3d-C cells were indeed much less proliferative (Fig. 1f, g). Within the EdU^−^ population, most were Pax7^+^MyoD^−^ for 2 + 3d-C cells, while the majority were Pax7^−^MyoD^+^ for 5d-U cells (Fig. 1h, Supplementary Fig. 4f). Furthermore, the level of phosphor-P38 MAP kinase (pP38) was lower in 2 + 3d-C cells than that in both 2d-U and 5d-U cells, consistent with the former being in the stem cell state (Supplementary Fig. 4g, h)^19^. Together, a ~55% compression on MuSCs enriches for a non-proliferative Pax7^+^MyoD^−^ state.

### Compressed MuSCs retain proliferation and differentiation potential

Notably, after removing the compression pillar, we determined the 2 + 3d-C cells could increase cell density in the ensuing days (Supplementary Fig. 5a, b). When they reached a cell density comparable to that of 5d-U cells (7-day post compression removal, 2 + 3d-C+7), we switched them into the differentiation medium and cultured for an additional 4 days (2 + 3d-C + 7 + 4). The 2 + 3d-C + 7 + 4 cells expressed myosin heavy chain (MF20) and fused as multinucleated myotubes. The percentage of MF20^+^ cells and the fusion index of de-compressed cells were similar to those of the 5d-U cells subjected to the same differentiation condition (5d-U + 4; Fig. 1i–k; Supplementary Fig. 5c). Together, these data indicate that mechanically compressing MuSCs to 4 μm cell height—similar to their physiological cell height in uninjured muscle—can efficiently drive them into a non-proliferative Pax7^+^MyoD^−^ state while retaining the potential for expansion and myogenic differentiation.

### Differentiation committed myogenic cells to regain MuSC fate after compression

We also assessed the effect of compression on MuSCs that had been cultured longer. After 7 days of culture (7d-U), Pax7^−^MyoD^+^ cells were the majority (>60%), while Pax7^+^MyoD^−^ cells were scarce. After applying compression for additional 3 days (7+3d-C), we found that the Pax7^−^MyoD^+^ fraction decreased substantially (to <10%). We did observe a significant increase of the Pax7^+^MyoD^−^ faction (~40%), though less than that in the 2 + 3d-C condition (Fig. 1l; Supplementary Fig. 5d). The decrease of the Pax7^−^MyoD^+^ fraction under 2 + 3d-C and 7+3d-C conditions (from their respective starting points) suggests that some Pax7^−^MyoD^+^ cells turn on Pax7 under compression.

### High axial tension and overall (azimuthal) tension are both required for MuSC fate

**Fig. 2:**
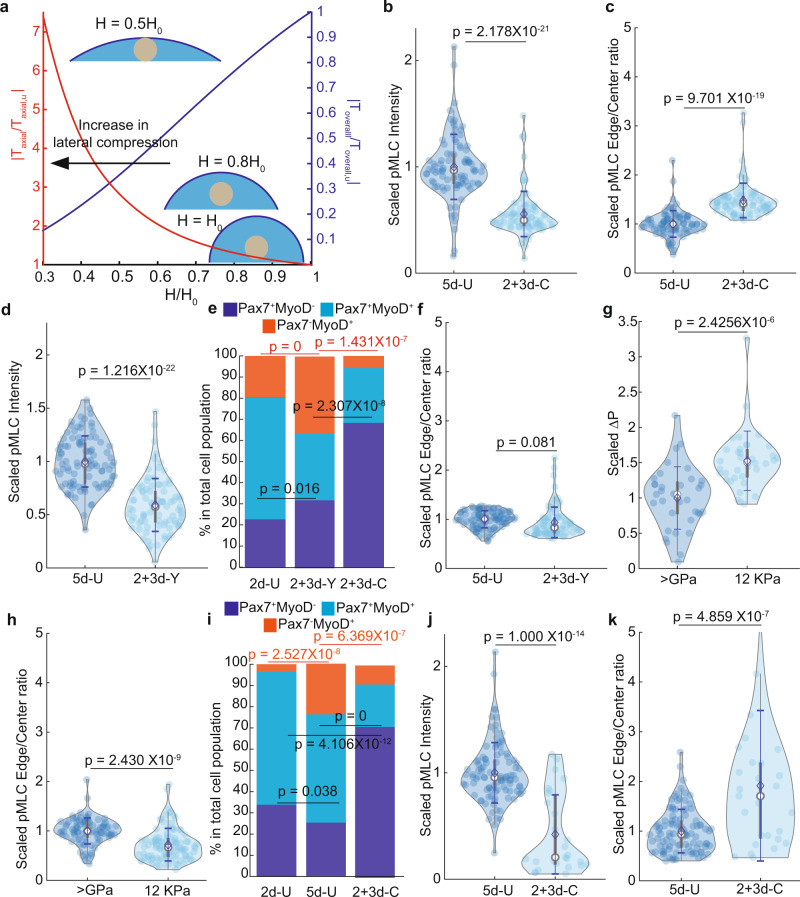
Tension analysis for MuSCs under different conditions. **a** Mathematical model prediction of change in average tension distribution in terms of compressive strain. **b** and **c** pMLC distribution of 5d-U and 2+3d-C cells. This is in terms of overall pMLC (**b**) and pMLC Edge/Center ratio (**c**) (*n* = 3 for both 5d-U and 2+3d-C cells. Each repeat was scaled with the mean value of its respective 5d-U; of total 86 cells for 5d-U and 84 cells for 2+3d-C). **d** and **f** pMLC distribution of 5d-U and 2+3d-Y cells. This is in terms of overall pMLC (**d**) and pMLC Edge/Center ratio (**f**) (*n* = 3 for both 5d-U and 2+3d-Y cells. Each repeat was scaled with the mean value of its respective 5d-U, of total 95 cells for 5d-U and 87 cells for 2+3d-C). **e** Cell fate comparison between 2d-U, 2+3d-Y, and 2+3d-C cell (2d-U cell fate data is the same as in Fig. 1c. *n* = 4 for 2+3d-Y, of total 268 cells; *n* = 3 for 2+3d-C cells, of total 77 cells). **g** Pressure comparison between 5d-U cells seeded on plastic (stiffness > GPa) and on 12 kPa hydrogel (*n* = 9 for plastic-seeded cells, of total 35 cells. *n* = 9 for 12 kPa hydrogel-seeded cells, of total 36 cells). **h** Scaled pMLC Edge**/**Center ratio of 5d-U cells seeded on plastic and on 12 kPa hydrogel (5d-U cells on plastic are the same as in (**b**) and (**c**). *n* = 3 for 5d-U cells seeded on 12 kPa hydrogel, of total 100 cells). **i** Cell fate comparison between 2d-U, 5d-U, and 2+3d-C cells seeded on 12 kPa hydrogel (*n* = 3 for 2d-U, of total 148 cells. *n* = 4 for 5d-U, of total 532 cells, *n* = 4 for 2 + 3d-C, of total 228 cells). **j** and **k** pMLC distribution of 5d-U and 2+3d-C cells seeded on 12 kPa hydrogel. This is in terms of overall pMLC (**j**) and pMLC Edge/Center ratio (**k**) (5d-U data is the same as in (**h**). Each repeat was scaled with the mean value of its respective 5d-U. *n* = 3 for 2+3d-C, of total 27 cells). Modeling results presented in (**a**) is up to second-order accuracy (see the “Methods” section under the subsection “Mathematical model for solving cell shape”). **b**–**h**, **j**, and **k** data were presented with mean$\pm$s.d. $p$-value was assessed with Student’s two-tail *t*-test using MATLAB. Data in **e** and **i** were presented with overall fractions. $p$-value was assessed based on a two-tailed Cochran–Mantel–Haenszel test using MATLAB. The comparison was considered significant if $p\le 0.05$. Representative images are shown in Supplementary Fig. 6.

Next, we examined the key change(s) in cell mechanical tension under compression. We first used a force-balance-based mathematical model^20,21^ to estimate the relative change of cellular tension along the two principal (azimuthal and axial) directions when the compression is applied (Supplementary Fig. 6a). Two assumptions in this model are: 1) cell volume is at a constant at any given cell height and 2) cell tension is linearly scaled with local cell geometry, i.e., the local radius of curvature^20,22^ (see the “Methods”). We quantified the cell volume of MuSCs by confocal imaging and found no significant changes before and immediately after compression, validating the first assumption (Supplementary Fig. 3c). The model predicts that, under compression, MuSCs experience a higher axial tension and a lower overall (azimuthal) tension as they become flattened and axially extended (Fig. 2a). A 4 μm height limitation, or ~55% of lateral compression to 2d-U cells predicts a ~60% reduction of overall tension and a ~2-fold increase in axial tension (Fig. 2a). To determine these changes, we probed for the phospho-myosin light chain (pMLC) level as a proxy for cell tension^23–25^. We evaluated the overall pMLC intensity (indicating overall tension) and the pMLC edge-to-center ratio (indicating relative axial tension; Methods). Compared to 5d-U cells, 2 + 3d-C cells had a significantly lower overall pMLC level and higher edge-to-center pMLC ratio (Fig. 2b, c; Supplementary Fig. 6b). Thus, experimental data support our modeling that mechanical compression causes lower overall but higher axial tension.

We further tested whether low overall and high axial tensions are both necessary to affect MuSC fate. We first reduced both overall and axial tensions by treating the cells with Y-27632 (which inhibits ROCK, an upstream effector of pMLC) after 2 days of culture and assaying them at day 5 (2 + 3d-Y cells). The 2 + 3d-Y cells had a ~50–60% reduction of overall pMLC level compared to 5d-U cells, similar to that of the 2 + 3d-C cells (Fig. 2d; Supplementary Fig. 6b). Atomic force microscopy (AFM) determined a ~50% reduction in 2 + 3d-Y cells’ cross-membrane pressure, Δ*P* (linearly scaled with overall tension^20,21^; see the “Methods” section), from that of 5d-U cells (Supplementary Fig. 6c), which supported pMLC level as a proxy for cell tension. Although 2 + 3d-C and 2 + 3d-Y cells had a similarly reduced overall pMLC level, the latter showed no increase of pMLC edge-to-center ratio, relative to 5d-U cells (Fig. 2f; Supplementary Fig. 6b). Interestingly, 2 + 3d-Y cells had a moderately higher and significantly lower Pax7^+^MyoD^−^ fraction than 2d-U and 2 + 3d-C cells, respectively, and a significantly higher Pax7^+^MyoD^−^ fraction than 5d-U cells (Fig. 2e, Supplementary Fig. 6d, e). Thus, lowering overall tension without increasing axial tension yielded only a slight increase in Pax7^+^MyoD^−^ cell fraction. Applying compression onto Y-27632-treated MuSCs resulted in substantial cell loss, precluding assessment. We next increased the overall cell tension by culturing MuSCs on Matrigel/fibronectin-coated 12 kPa hydrogel^25^. 5d-U cells on 12 kPa had a ~60% higher Δ*P* than those on plastic (determined by AFM; Fig. 2g). The pMLC edge-to-center ratio of cells on 12 kPa was ~10–15% lower than that of cells on plastic (Fig. 2h), reflecting ~30% higher absolute axial tension after taking the increased total tension into account. In both 2d-U and 5d-U settings, MuSCs on 12 kPa had a higher portion of Pax7^+^MyoD^−^ than those on plastic (Supplementary Fig. 6f, g), consistent with the report that 12 kPa is better in maintaining self-renewal divisions of MuSCs^10,11^. However, MuSCs cultured on 12 kPa still lose Pax7^+^MyoD^−^ cells from 2d-U to 5d-U (Fig. 2i). Thus, simultaneously elevating overall and axial tensions were insufficient in restoring Pax7^+^MyoD^−^ fate. By contrast, on 12 kPa, the 2 + 3d-C cells showed a lower overall level and a higher edge-to-center ratio of pMLC compared to 5d-U cells. This followed a similar trend of pMLC distribution from 2 + 3d-C to 5d-U cells on plastic (Fig. 2j, k; Supplementary Fig. 6h, i). Importantly, the Pax7^+^MyoD^−^ fractions of 2 + 3d-C cells, on plastic and 12 kPa, were also similar (Fig. 2i; Supplementary Fig. 6f, g) and were significantly higher than those in respective 2d-U and 5d-U cells. Together, these results indicate that the low overall tension and high axial tensions concomitantly achieved by mechanical compression are most effective in supporting the Pax7^+^MyoD^−^ fate—even though the basal chemistries between the ECM-coated 12 kPa hydrogel and plastic surface are different. By extension, the flattened and extended shape of quiescent MuSCs in vivo (Supplementary Fig. 1a–c) implies that they experience higher axial tension and lower overall tension by mechanical compression.

### Compressed MuSCs have higher levels of Notch signaling

**Fig. 3:**
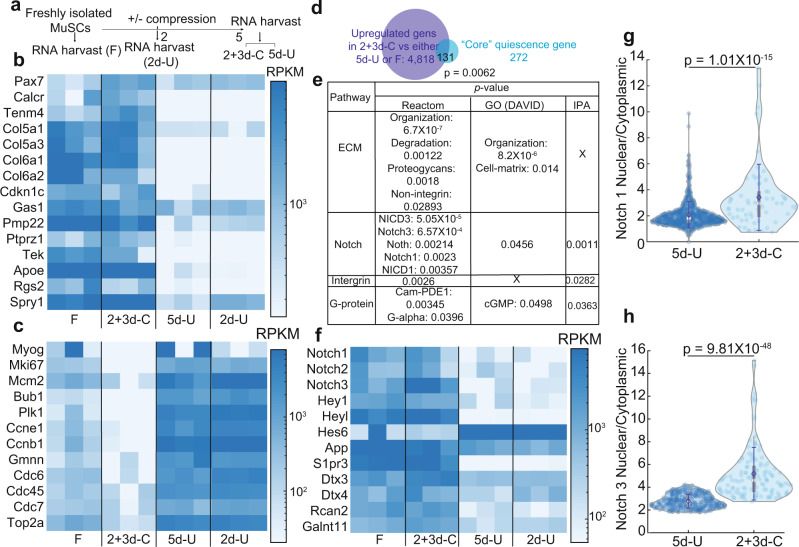
Transcriptome comparison between 2+3d-C, 5d-U, and F cells. **a** Experimental setup. **b**, **c**, **f** Reads Per Kilobase of transcript, per Million mapped (RPKM) readings of MuSCs’ quiescence and stemness (**b**), activation and differentiation (**c**), and Notch downstream genes (**f**). **d** Comparison between upregulated genes in 2+3d-C cells and “core” quiescence genes. **e** Pathway analyses on overlapped “core” quiescence genes. **g** and **h** Notch1 (**g**) and 3 (**h**) nuclear/cytoplasmic ratio comparison between 5d-U and 2+3d-C (*n* = 3 for each experimental set in RNA-seq; (**g**) *n* = 4 for 5d-U (+DMSO), of total 695 cells. *n* = 4 for 2+3d-C (+DMSO), of total 61 cells. **h** *n* = 5 for 5d-U (+DMSO), of total 291 cells. *n* = 3 for 2+3d-C (+DMSO), of total 124 cells). **b**, **c**, **f** RPKM readings, as well as respective *p-*values, were calculated by DESeq2.r package and MATLAB bioinformatics package. All presented genes have $p\le 0.05$. *p-*value for overlapping genes (**d**) was calculated by Fisher exact. *p-*value for each enriched pathway (**e**) was calculated respective pipelines. If $p > 0.05$, a “X” is marked. **g**, **h** data were presented with mean $\pm$s.d. *p-*value was assessed with Student’s two-tail *t*-test using MATLAB. The comparison was considered significant if $p\le 0.05$. Representative images for **g**, **h** are shown in Supplementary Fig. 8a, b. Normalized reads for **b**, **c**, **f** are in Supplementary Tables 1–3.

To uncover the molecular signature of compressed MuSCs, we interrogated the RNA-seq data among F, 2d-U, 2 + 3d-C, and 5d-U MuSCs (Fig. 3a). Principal component analysis indicated that F and 2 + 3d-C MuSCs were more similar than 2d-U and 5d-U cells (Supplementary Fig. 7a). The similarity between 2d-U and 5d-U cell populations likely reflected a large Pax7^+^MyoD^+^ cell fraction in both (Fig. 1c). Genes associated with MuSC quiescence^10,26–30^, particularly *Pax7, calcitonin receptor (Calcr)*, and *collagen 5a1*&*3 (Col5a1, Col5a3)*, were upregulated in 2 + 3d-C cells (Fig. 3b, Supplementary Table 1), despite the presence of non-Pax7^+^MyoD^−^ cells. A high level of *Pax7* is linked to MuSC stemness^31^, and *Calcr* and *Col5a1*&*3* are critical for MuSC quiescence^30,32^. In contrast, genes related to MuSC activation, proliferation or myogenic differentiation^26,28^ were upregulated in 2d-U and 5d-U cells (Fig. 3c, Supplementary Table 2). Together with the EdU data (Fig. 1h), 2 + 3d-C Pax7^+^MyoD^−^ cells represent a cell fate/state mimicking quiescent MuSCs^28,29^.

Recent studies documented substantial differences between F and in situ*-*fixed MuSCs, by assessing either steady-state or nascent mRNA transcripts^28,29^. Genes upregulated in fixed MuSCs were not identical between steady-state and nascent transcriptomes but had an overlap of 272 genes. We used these genes as core quiescent genes (see the “Methods” section) to understand the quiescent 2 + 3d-C MuSCs (Supplementary Fig. 7b). Pathway analysis of the core quiescent genes by IPA and Reactome showed enrichment for Notch, Hedgehog, and G-protein-related signaling (Supplementary Fig. 7c). The upregulated genes in 2 + 3d-C MuSCs (compared to both 2d-U and 5d-U cells) showed enrichment for Notch signaling, as well as actin-related cell mechanics and cell–ECM-related interactions (likely due to compression), by IPA and GO (DAVID) (Supplementary Fig. 7d). One hundred and thirty-two of the core quiescent genes were upregulated in 2 + 3d-C MuSCs compared to 5d-U or F cells (Fig. 3d; $p=0.0062$, Fisher’s exact). Pathway analysis of the 131 genes by aforementioned informatic tools all showed Notch signaling enrichment (Fig. 3e). We obtained similar outcomes by analyzing upregulated genes in 2 + 3d-C cells relative to 2d-U cells (Supplementary Fig. 7e, f). Direct downstream genes of Notch, such as *Her* genes^26,30,33^, were indeed upregulated in 2 + 3d-C cells (Fig. 3f, Supplementary Table 3). To demonstrate Notch activation by compression, we examined the cellular localization of Notch 1&3 in MuSCs, as Notch1 plays a role in maintaining MuSCs stemness while Notch3 orchestrates MuSCs to return to quiescence^34,35^. Compared to 5d-U cells, 2 + 3d-C cells displayed nuclear enrichment of Notch1&3 (Fig. 3g, h; Supplementary Fig. 8a, b), providing evidence for Notch activation.

### Compression-induced Notch signaling is independent of Adam10/17

**Fig. 4:**
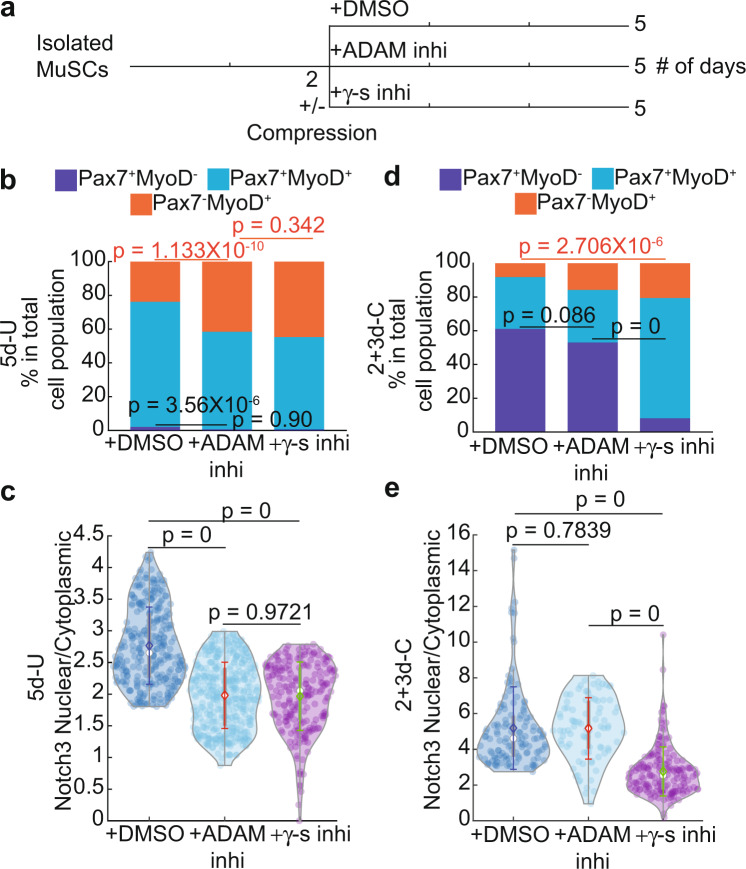
Notch nuclear enrichment assay and cell fate evaluation after blocking S2 (+ADAM inhi) or S3 (+γ-s inhi). **a** Experimental setup. **b** and **c** 5d-U cells’ fate (**b**) and Notch3 nuclear/cytoplasmic ratio (**c**) comparison after between control (+DMSO) and blocking S2 or S3 cleavage (**b** *n* = 7 for +DMSO, of total 1303 cells; *n* = 3 for +ADAM inhi, of total 332 cells; *n* = 3 for +γ-s inhi, of total 596 cells. **c** +DMSO data is same as in Fig. 3j. *n* = 4 for +ADAM inhi, of total 423 cells. *n* = 3 for +γ-s inhi, of total 215 cells). **d** and **e** 2+3d-C cells’ fate (**d**) and Notch3 nuclear/cytoplasmic ratio (**e**) comparison between control (+DMSO) and blocking S2 or S3 cleavage (**d** *n* = 7 for +DMSO, of total 280 cells; *n* = 5 for +ADAM inhi, of total 183 cells; *n* = 4 for +γ-s inhi: of total 715 cells. (**e**) +DMSO data is the same as in Fig. 3j. *n* = 3 for +ADAM inhi, of total 88 cells. *n* = 3 for +γ-s inhi, of total 169 cells). Data in **b** and **d** was presented with an overall fraction. $p$-value was assessed based on a two-tailed Cochran–Mantel–Haenszel test using MATLAB. (**c**) and (**e**) data was presented with mean $\pm$s.d. $p$-value was assessed with Kruskal–Wallis tests using MATLAB. The comparison was considered significant if $p\le 0.05$. Representative images are shown in Supplementary Fig. 8c–f.

We next investigated the cellular mechanism underlying compression-induced Notch activation. Canonical Notch activation requires binding of the ligand Delta (Dll or Dll-related members in mammal cells) from neighboring cells. Pulling force on Dll by endocytosis of neighboring cells causes a conformational change of the juxta-membrane negative regulatory region (NRR) domain of Notch for S2-cleavage by ADAM10 or 17. Intramembrane S3-cleavage by γ-secretase then follows and initiates Notch intracellular domain’s nuclear enrichment, affecting downstream gene expression^36^. As we did not provide exogeneous Dll with a pulling force, the canonical mode was unlikely to mediate nuclear-enriched Notch1&3. In addition, 2 + 3d-C MuSCs were singly isolated and mostly non-proliferative, making a low probability for Dll-Notch signaling via cell–cell contact^37^. We therefore considered a Dll-independent cell-autonomous mode of Notch activation by compression. If so, S2-cleavage might not be needed, while the S3-cleavage should still be required for Notch nuclear enrichment. To test this, we inhibited S2-cleavage by blocking ADAM10 and 17 with GI254023X and TAPI-0, respectively, and S3-cleavage by blocking γ-secretase with DAPT. We compared Notch3 nuclear enrichment and cell fate of 5d-U and 2 + 3d-C MuSCs under these pharmacological treatments to the mock-treated controls (Fig. 4a; see the “Methods” section). Inhibition of either S2- or S3-cleavage increased the Pax7^−^MyoD^+^ cell fraction and decreased nuclear Notch3 to a similar level in 5d-U MuSCs (Fig. 4b, c; Supplementary Fig. 8c–f). Compression-induced increases of Pax7^+^MyoD^−^ cell fraction and nuclear Notch3 were only abolished by inhibiting S3-cleavage (Fig. 4d, e; Supplementary Fig. 8c–f). Since blocking S2 or S3-cleavage achieved similar results for 5d-U cells, the difference in these treatments found in 2 + 3d-C cells implied that the canonical Dll-dependent S2-cleavage of Notch was not critical for MuSC stemness under compression. We suggest that compression-induced tension distribution triggers a conformational change of NRR for a cryptic cleavage, i.e., not mediated by ADAM10/17, near the S2 site to activate Notch. Altogether, our results support a mechanism in which the combined low overall and high axial tensions from compression create a membrane topology permissible for eventual S3-cleavage of Notch in the MuSC.

## Discussion

The concept of activated MuSCs returning to quiescent under apical compression helps explain their activity in vivo. During the injury-regeneration cycle, MuSCs residing over a damaged myofiber segment lose apical contact and become activated for regeneration^6^. As newly regenerated muscle fibers form and undergo hypertrophic growth, their compression force onto MuSCs increases. This can serve as a timed-mechanical control as regenerated muscles reach their original size and mechanical properties, instructing Pax7^+^MyoD^+^ and Pax7^−^MyoD^+^ populations to return to their quiescent stem cell state. Such control prevents overt regeneration and maintains a relatively constant MuSC-to-muscle ratio after each injury-regeneration cycle, i.e., proportional regeneration. A similar scenario likely applies to establishing the initial quiescent MuSC pool when postnatal muscles reach a mature size to exert sufficient compression force to instruct MuSC quiescence. This principle may be extended to the scaling between MuSCs and muscles during adaptive muscle size changes, such as exercise and aging. Importantly, our in vitro-engineered niche activates the Notch signaling pathway utilized for MuSC quiescence in vivo^30,35^. Synthesizing the known signaling pathways and our findings herein, we re-interpret that Cadherins act to decrease overall tension by mediating apical adhesion with the myofiber^38^, and Wnt-Rho signaling orchestrates tension re-distribution^39^ (Supplementary Fig. 7b). Both measures are accomplished by compression to activate Notch for MuSC quiescence. Furthermore, myofiber-expressed Dll has been implicated in the signaling of MuSC quiescence^40^ and shown to be localized at MuSCs’ cell edge^41^, a predicted high axial tension region caused by compression. We propose that Dll-induced and Dll-independent tension-sensing Notch signaling co-exist to assure the steady state MuSC quiescence over long-term muscle use, i.e., mechanical loading of muscle fibers^42^. Our results differ from the dysregulated, cell-autonomous activation of Notch caused by cancer-promoting mutations in Notch(es)^43^ or by mutations in genes regulating endo-lysosomal trafficking of Notch^44,45^. Whereas we addressed a previously unexplored compression force in stem cell quiescence, mechanical stimuli such as ECM elasticity, stretching, and osmolarity, are known to stimulate the proliferation or differentiation of stem cells in various tissues, including the skeletal muscle^11,14,46–48^. Thus, it stands to reason that distinct quiescent and stimulatory microscopic niche mechanics defined by the macroscopic mechanical properties of a resident tissue are sensed by stem cells for tissue homeostasis.

## Methods

### Manufacturing microfluidic compression device

The compression device is fabricated using a soft-lithography technique^49^. The first step is to etch a silicon wafer with UV light to a specific pattern and depth (used as a mold).

The compression pillar pattern was designed by AutoCAD (Supplementary Fig. 2a, b) and printed onto a photomask. Each pattern group had a designed size comparable to the area of one well in a 24-well plate, and one photomask contained eight pattern groups. This photomask allows the UV light to etch only at the designated pillar regions (Supplementary Fig. 2b). The wafer (Wafer World) is spun-coated with SU8 3005 photoresists (3000 rpm, 1 min) at a specific mixing ratio, which allows the UV to etch 4 μm into the wafer. We then align the photomask with the wafer using EV620 Double-Sided Mask Aligner and expose it to UV.

PDMS Sylgard 184 silicone elastomer and curing agent (DOW) were thoroughly mixed at 10:1 ratio and vacuum degassed next. We spun-coat a thin layer of PDMS (500 rpm, 15 s) onto the mold and baked it at 70 °C until the PDMS cured (~25 min). The cured PDMS film contained protruded-out pillars, with a height of ~4 μm, following the caved-in pillar pattern on the mold (referred to as pillar-side) (Supplementary Fig. 2c). Then, the PDMS is then carefully peeled off from the mold and attached to a cover glass at the non-pillar side.

### Mathematical model for solving cell shape

The change in local cellular curvature and apical tension due to externally applied mechanical compression can be recapitulated by using a force-balance-based mathematical model:^20,21,25^

1 $$\varDelta P{\left(\nabla \cdot {{{{{\bf{n}}}}}}\right)}^{-1}-T=0$$

in which **n** is the local unit normal vector. $\nabla$ is operating at the local cell membrane surface. *T* is cell tension, and $\varDelta P$ is the intracellular-to-extracellular hydrostatic pressure difference. By geometric definition, $\left(\nabla \cdot {{{{{\bf{n}}}}}}\right)$ equals to the trace of curvature tensor: $\nabla \cdot {{{{{\bf{n}}}}}}={c}_{1}+{c}_{2}$, which describes the local shape of the cell (${c}_{1}$ is the curvature along the azimuthal direction and ${c}_{2}$ is the curvature along the axial direction). We numerically solved Eq. (1) under the constant volume constraint by discretizing it to second-order accuracy. We programmed the solver using MATLAB code.

Assuming symmetric shape, we parameterized the cell surface using cell local radius, *R* and azimuthal angle ⎝ (Supplementary Fig. 6a). Equation (1) can be written as

2 $${(\nabla \cdot {{{{{\bf{n}}}}}})}^{-1}={\left(\underbrace{\frac{2{R}^{{\prime} 2}+{R}^{2}-R\frac{{d}^{2}R}{d{\theta }^{2}}}{{\left({R}^{2}+{\frac{dR}{d\theta }}^{2}\right)}^{1.5}}}_{{c}_{1}}+\underbrace{\frac{1-\frac{\frac{dR}{d\theta }}{R}\cot \theta }{\sqrt{{R}^{2}+{\frac{dR}{d\theta }}^{2}}}}_{{c}_{2}}\right)}^{-1}=\frac{T}{\Delta P}$$

Two boundary conditions are required to solve this equation. First, under compression, the cell height is fixed at *H*: $R\left(\theta =0\right)=H$. Second, based on the symmetric condition: $\frac{{{\mathrm {d}}R}}{{\mathrm {d}}\theta }\left(\theta =0\right)=0$. We close the system by assuming: $T \sim k\left(\frac{1}{c1}+\frac{1}{c2}\right)$ and the cell retains a constant volume, *V*_0_, right when the vertical compression is applied (*V*_0_ is uncompressed cell volume; Supplementary Fig. 3c). Supposed uncompressed cell has a hemisphere shape with radius of ${R}_{0}$ (~8.7 μm; Supplementary Fig. 3b):

3 $$V=\pi {\int }_{\!\!\!0}^{\frac{\pi }{2}}{R}^{2}\theta \left(R\sin \sin \theta -\frac{{{\mathrm {d}}R}}{{\mathrm {d}}\theta }\cos \cos \theta \right){\mathrm {d}}\theta ={V}_{0}=\frac{2}{3}\pi {R}_{0}^{3}$$

Numerically, we discretized $R\left(\theta \right)$ with finite angle increment ${\mathrm {d}}\theta =\frac{\pi }{1000}$. We expand the expression for *R* using Taylor series and approximate the first and second derivative to the second-order accuracy:

4 $$\frac{{dR}}{d\theta }(\theta ) 	= \frac{R\left(\theta +d\theta \right)-R\left(\theta -d\theta \right)}{2d\theta }+O\left(d{\theta }^{2}\right) \\ \frac{{d}^{2}R}{d{\theta }^{2}}(\theta ) 	= \frac{R\left(\theta +d\theta \right)+R\left(\theta -d\theta \right)-2R\left(\theta \right)}{d{\theta }^{2}}+O\left(d{\theta }^{2}\right)$$

By solving discretized Eq. (2) under aforementioned constrain/assumption (volume error tolerance was set at 1%: $\left|\frac{V-{V}_{0}}{{V}_{0}}\right|\le 0.01$), we can solve for cell shape: ${c}_{1}$ and ${c}_{2}$.

Substituting ${c}_{1}$ and ${c}_{2}$ to: $T \sim k\left(\frac{1}{c1}+\frac{1}{c2}\right)=\frac{k}{{c}_{1}}+\frac{k}{{c}_{2}}$: We define: $\frac{k}{{c}_{1}}$ as azimuthal (overall) tension: ${T}_{{{\mathrm {overall}}}}$ and $\frac{k}{{c}_{2}}$ as axial tension, ${T}_{{{\mathrm {axial}}}}$.

### Atomic force microscopy (AFM)

AFM experiments were done with a silicon nitride (SiN) cantilever and tips with a nominal spring constant of 0.01 N/m (Bruker, USA) on an MFP3D (Asylum Research, USA) instrument. Thermal fluctuation method was used to calibrate the stiffness of the cantilever before every experiment^50^. To measure the apical stiffness, cells were indented using contact mode to get force–displacement curves (Supplementary Fig. 9a, c). The cell-to-cantilever contact area was estimated to be 1 μm^2^. All data processing was done using Igor pro software (Wavemetrics, USA). The indentation was done by indenting the cells with depths of no more than 1.5 μm so that it would fall into the category of “small indentation”. The indentation increment was set so that there were 400 indentation steps for the depth–force curve for each cell. The mathematical model used to approximate the overall tension from AFM data was similar to Eq. (1) but under different conditions. The details were described under the subsection “Approximating overall tension from AFM data using a mathematical model” and the example indentation strain–stress curves with their respective fitting are shown in Supplementary Fig. 9.

### Approximating overall tension from AFM data using a mathematical model

Because of the small-strain indentation (<10% of total cell height) and the small contact area between the AFM cantilever and the cell surface, the cell remained at a constant volume and a relatively constant intracellular-to-extracellular pressure difference. Cell membrane surface parametrization was described in the previous section (Supplementary Fig. 6a). The mechanical energy function, *E*, describes the cellular shape change in response to extracellular force.

5 $$E={\int }_{\!\!\!\!A}T{\mathrm {d}}A-{\int }_{V}\Delta P\,{\mathrm {d}}V$$

the functional derivative of *E* (Eq. (5)) in terms of the cell’s apical area, *A*,$\frac{\delta E}{\delta A}$, is the classic normal force shown as the left-hand side of Eq. (1).

To satisfy the constant volume constrain (i.e., the indented volume, $V$, equals to the un-indented volume, ${V}_{0}\left)\right.$, we added a LaGrange multiplier term to the mechanical energy: $E=E+{\lambda }_{0}({V}_{0}-V)$. Setting $\frac{\delta E}{\delta A}$ to 0:

6 $$\varDelta P{\left(\nabla \cdot {{{{{\bf{n}}}}}}\right)}^{-1}-T-{\lambda }_{0}\frac{\delta V}{\delta A}=0$$

$\frac{\delta V}{\delta A}=\frac{\delta V}{\delta R}{\left(\frac{\delta A}{\delta R}\right)}^{-1}$, which is a complicated function. Because of the small strain indentation condition, $\frac{\delta A}{\delta R} \sim {r}_{0}$; in which ${r}_{0}$ is cell’s spreading radius (Supplementary Fig. 9b). $\frac{\delta V}{\delta R}=\pi \left(3{R}^{2}\theta +2R{R}^{\prime}\theta \cos \cos \theta \,\theta \right)$ according to Eq. (3). We define a new constant: $\lambda =\pi \frac{{\lambda }_{0}}{{r}_{0}}$. Equation (6) can then be approximated as

7 $$\varDelta P{\left(\nabla \cdot {{{{{\bf{n}}}}}}\right)}^{-1}-T-\lambda \left(3{R}^{2}\theta +2R\frac{\partial R}{\partial \theta }\theta \cos \cos \theta \,\theta \right)=0$$

Applying boundary conditions: $R\left(\theta =\frac{\pi }{2}\right)={r}_{0}$ and $R\left(\theta =0\right)={r}_{0}-H$ ($H$: indentation depth; Supplementary Fig. 9b) and combining Eq. (7) with Eq. (3), we can solve for indented cell shape, $\lambda$, and $\frac{T}{\varDelta P}$.

Taking Eq. (7) at the cantilever–cell membrane contact surface, the feedback force per unit area from the AFM cantilever is

8 $$F=\left(\varDelta P+\frac{\lambda V}{A}\right)-T\left(\nabla \cdot n\right){{{{{{\rm{|}}}}}}}_{\theta =\frac{\pi }{2}}$$

The physical meaning of $\lambda V$ is the added volumetric force to maintain a constant volume (i.e., incompressible pressure). We adjusted the pressure difference with the added volume-constrain force: ${P{{\hbox{'}}}}=\varDelta P+\frac{\lambda V}{A}$

To numerically solve this set of equations, we expand the geometry of the cell using Fourier Series:

9 $$r={r}_{0}+\mathop{\sum} \limits_{n}{a}_{n}{{\cos }}(n\theta )+\mathop{\sum} \limits_{n}{b}_{n}{{\sin }}(n\theta )$$

Considering of boundary conditions discussed above, we have: ${\sum }_{n}{a}_{n}{{\cos }}\left(\frac{n\pi }{2}\right)+{\sum }_{n}{b}_{n}{{\sin }}\left(\frac{n\pi }{2}\right)=0$, which satisfies the adhesion boundary condition. ${\sum }_{n}{a}_{n}=H-{r}_{0}$ satisfies the constant cell height. The coefficients of this expansion should give a cell geometry that satisfies Eq. (3) and $\frac{{\rm {d}}\lambda }{{\rm {d}}\theta }=0$, which is derived from Eq. (7). *R* is expanded up to the second term of the Fourier series, which gives the four parameters.

With the best fit, we calculated the theoretical $\frac{F}{{P{{\hbox{'}}}}}$ in terms of the indentation strain (Supplementary Fig. 9d–f, green line). This means, regardless of cell type and mechanical condition, any indentation strain–stress curve should collapse with the theoretical indentation strain-$\frac{F}{{P{{\hbox{'}}}}}$ curve with a scaling constant on the measured stress. This scaling constant is then the estimated $\frac{1}{{P{{\hbox{'}}}}}$ for that specific cell. Example indentation strain–stress curves for 5d-U cells on plastic and 12 kPa hydrogel and 2 + 3d-Y cells (Supplementary Fig. 9c) indeed fit the theoretical strain-$\frac{F}{{P{{\hbox{'}}}}}$ curve (Supplementary Fig. 9d–f), which gives their respective $\frac{1}{{P{{\hbox{'}}}}}$ for the respective condition. We can then estimate the overall tension from the fitted pressure: ${T}_{{{\rm {overall}}}} \sim {P{{\hbox{'}}}\; L}.$ $L$ is the typical length scale of the cell, usually in the order of the un-indented cell height, ${r}_{0}$. Even though cell heights between 5d-U cells on plastic or 12 kPa or 2 + 3d-Y cells may differ, they are in a similar order of magnitude. Therefore, using fitted ${P{{\hbox{'}}}}$ to approximate overall tension reflects the general trend of overall tension between these three conditions. Specifically, regardless of whether the cells are seeded on 12 kPa hydrogel or plastic, a 4 μm compression decreases the overall pMLC level by 50% (Fig. 2b, j). This indicates that the cellular height of plastic-seeded cells is similar to that of 12 kPa hydrogel-seeded cells.

### Isolation and culturing of MuSCs

All experimental procedures for the mouse were approved by Carnegie Institutional Animal Care and Use Committee (IACUC). Muscle satellite cells were isolated from Pax7-ZsGreen mice^17^ (3–6 months old) and subjected to FACS (BD FACSAria III, controlled by BD FACSDiva software) using a protocol modified from Liu et al.^51^.

The ZsGreen^+^ cells were isolated according to the gate settings shown in Fig. 1a and collected into wash medium, containing 10% horse serum (HS; Gibco) in Ham’s F-10 medium (Thermo Fisher). The gating was determined by plotting FITC-A (green) fluorescent signal against a negative fluorescent signal (e.g., PE-A) for the cells (in log_10_ scale, Fig. 1a). The cell population that has a high FITC-A signal (i.e., away from the main autofluorescent line, labeled in red, Fig. 1a) was collected as Pax7^+^ population (~2% of total cell population, labeled in green, Fig. 1a). To determine sorting efficiency, 150 μL of sorted cell solution was spun onto a glass slide using Cytospin^TM^ centrifuge with cytofunnels and cytoclips (Thermo Scientific). The wetted area (on which the cells were landed) from the glass slide was marked by hydrophobic pen (Vector Laboratories H-400). They were then probed for Pax7 and MyoD expression using the procedures described under the subheading of Immunofluorescence.

Isolated cells were seeded in a 24-well plate with plastic (Falcon) or 12 kPa hydrogel (Matrigen) bottom, unless specified otherwise, at a density of 3000–4000 cells per well. For the AFM experiment, cells were seeded in plastic or 12 kPa hydrogel dishes of 60 or 100 mm diameter at a similar cell density. All the wells and dishes were pre-coated with Matrigel (Corning) and fibronectin (Sigma-Altrich). Matrigel stock solution was provided by the manufacturer at 13 mg/mL, and Fibronectin stock solution was prepared at 1 mg/mL in 1X phosphate-buffered saline (PBS, GIBICO). They were diluted into Dulbecco’s modified Eagle’s medium formulation (DMEM, GIBCO) to a final concentration of 1.3 mg/mL of Matrigel and 25 μg/mL of fibronectin as the coating solution. We applied 0.3 mL of coating solution to each well (and 1.5–3 mL per 60 or 100 mm dish) and incubated in a standard tissue culture incubator (37 °C and 5% CO_2_) for 1 h before seeding the cells. Seeded cells were cultured in growth medium (with or without compression) under standard conditions (37 °C, 5% CO_2_) for the duration specified in each set of experiments. The growth medium contains 1% penicillin–streptomycin (PS, Gibco), 0.01% of Fungin (InvivoGen) and Plasmocin (InvivoGen), 0.1% of chicken embryo extract (M.P. Bimedicals), 10 ng/mL fibroblast growth factor (FGF, BD Bioscience), 5% of HS and 20% of fetal bovine serum (FBS, Gibco) in DMEM. The differentiation media contains 10% HS in DMEM.

### Drug treatments

All drug treatments on the cell culture took place after 2 days of culturing and lasted for 3 more days, as a direct comparison with 2 + 3d-C and 5d-U cells. The concentration for Y-27632 (Tocris, *K*_i_ = 0.14–0.22 and 0.3 μM for inhibiting ROCK1 and 2, respectively) was 25 μM. This was prepared by diluting 100 mM Y-27632 stock solution (in 1X PBS, recommended by the manufacturer as maximum concentration) in growth medium (1:4000 dilution). We chose 25 μM for Y-27632 treatment so that the average decrease of overall pMLC level in 2 + 3d-Y cells from 5d-U cells was similar to that of 2 + 3d-C cells. A similar concentration of 1X PBS was added to the control sets for both 5d-U and 2 + 3d-C cells (Growth medium + 0.025% 1X PBS). The working concentration for DAPT (Selleckchem.com, IC_50_ = 20 nM) is 10 μM diluting from 100 μM DAPT stock solution in growth medium with 8% DMSO (Sigma). The working concentration for DAPT on different types of cells ranges from 0.5 to 100 μM, according to the manufacturer. We chose 10 μM in that a 40–60% decrease of Notch3 nuclear-to-cytoplasmic ratio was observed for 5d-U cells. Using higher concentrations than 10 μM resulted in substantial cell loss after 3-day incubation. We choose the working concentrations of 3.5 and 30 μM for GI254023X (Sigma, IC50 = 5.3 nM) and TAPI0 (Tocris, IC_50_ = 50–100 nM) (ADAM 10 and 17 inhibitors), respectively, diluting from stock solution containing 35 μM of GI254023X and 300 μM TAPI in growth medium with 8% DMSO (1:10 dilution). These working concentrations were referenced from the published studies^52,53^. We adjusted the working concentrations so that the resulting Notch3 nuclear-to-cytoplasmic ratio for 5d-U cells was at the similar level compared to that of DAPT-treated 5d-U cells (Fig. 4c). The control (untreated) sets for both 5d-U and 2 + 3d-C cells were mocked-treated with growth medium containing 0.8% DMSO from day 2.

### Immunofluorescence

Unless specified otherwise, all steps were performed at room temperature. After removing the culture media, cells were fixed by adding 4% paraformaldehyde (Electron Microscopy Sciences; in 1X PBS) to the cell culture. For compressed cells, the compression device was removed before fixing. The cells were fixed and permeabilized (by 0.5% Triton in 1X PBS) for 10 min each. They were then blocked in 100% normal goat serum (NGS; GIBICO) for 1 h. Primary antibodies diluted in NGS were applied to each sample. They were rocked at 4 °C overnight. The samples were then washed twice with 0.1% Triton (in 1X PBS), treated with 4’, 6-diamidino-2-phenylindole (DAPI, 1:1000 dilution) plus appropriate secondary antibodies (diluted in NGS), and rocked for 1 h protected from light. They were then washed three times with 0.1% Triton (in 1X PBS) prior to imaging. The negative control samples for each 24-well plate were subjected to the same procedure in parallel without primary antibodies to the specific antigen.

All secondary antibodies were diluted in 100% NGS, with 1:1000 ratio. We used mouse IgG1 Pax7 primary antibody (Supernatant from hybridoma, DSHB) with 1:5 dilution ratio. The corresponding secondary antibody was Alexa flour goat anti-bouse IgG1 488 or 647 (Invitrogen). Primary antibody for MyoD was mouse IgG2b (Santa Cruz), with 1:250 dilution ratio. The corresponding secondary antibody was Alexa Flour goat anti-mouse IgG2b 568 or 647 (Invitrogen). Primary antibodies for Notch1 (Abcam), Notch3 (Abcam), and pMLC (Cell Signaling) were rabbit IgG, with dilution ratios of 1:200, 1:200, and 1:100 respectively. Corresponding secondary antibodies were Alexa Flour goat anti-rabbit IgG 647 or 488 (Invitrogen). Primary antibody for pP38 (Cell Signaling) was rabbit IgG, with dilution ratios of 1:100. Corresponding secondary antibody was Alexa Flour goat anti-rabbit IgG 647. Primary antibody for MF-20 was mouse IgG2b (Supernatant from hybridoma, DSHB), with 1:250 dilution ratio. The corresponding secondary antibody was Alexa flour Goat anti-mouse IgG2b 568.

### Epi-fluorescent Imaging

Fluorescent images were taken by a HAMAMATSU camera (Model C13440) mounted on a Nikon Ti2 E800 Scope under a ×20 air long-range objective, using the Nikon Element software. Images were acquired at the resolution of 2044 pixels × 2048 pixels (1 pixel = 0.23 μm). Digital images for each designated color channel were captured at the same exposure time throughout the same plate for quantitative comparison by mean pixel intensity. DAPI images were used to trace the nuclear contour, and bright field images were used to define cell boundaries.

### Quantitative imaging analysis

Cell boundary tracing and quantitative imaging analysis were performed using the MATLAB image analysis package. We do not include cell clusters in our quantification. Cell boundaries in the bright field images have sharp and distinguishable local intensity alteration regions (i.e., high-contrast contours; Supplementary Fig. 10a). These high-contrast contours were captured by 2D Gaussian smoothing filter (MATLAB built-in image analysis package, with standard deviation of 0.5) and were used to generate cell boundary binary mask by filling up any holes within the contour (Supplementary Fig. 10a). DAPI signals were traced and used to generate a binary mask for the nucleus (Supplementary Fig. 10b). Fluorescent intensities of each color channel for a specific target antigen were determined based on the cell or nuclear boundary masks for level and sub-localization. Specifically, each cell boundary was dilated to 10 and 25 pixels away, and the mean fluorescent intensity of the region between these two dilated boundaries (the region between yellow and blue dotted line: Supplementary Fig. 10c, d) was used as local background intensity for a given cell (Green shaded region: Supplementary Fig. 10d). The local background intensity was subtracted from the fluorescent pixel intensities within that cell/cell nucleus. This background subtracted value was then summed up and divided by the area (cell or nucleus) of interests to calculate the mean pixel intensity, which was used as present the expression level of target antigens in the cell/cell nucleus. Furthermore, the fluorescence intensity distribution of the negative control dish (treated without the targeted primary antibodies) was used to approximate the “cutoff” fluorescence intensity value for the positive signals (Supplementary Fig. 10e).

To determine the cellular distribution pattern of phosphor-Myosin Light chain (pMLC), the ratio of the relative pMLC level close to the cell edge verses towards the cell center was used. To define the “cell-edge” region, the traced cell boundary (Red solid line: Supplementary Fig. 10d) for each cell was objectively eroded in at a distance that was close to ~1/5 of average cell boundary-to-cell nucleus distance (Black dotted line: Supplementary Fig. 10d). The region within the eroded boundary is thus counted as cell-center region (Brown shaded region: Supplementary Fig. 10d), while the region between the cell boundary and the eroded boundary is considered as cell-edge region.

### Confocal imaging

Cells were cultured with CellBrite 488 (Biotium, 1:1000 dilution in growth media) membrane dye for 24 h prior to confocal imaging. We used a Zeiss LSM 800 confocal microscope equipped with a ×40 oil-immersion objective to take confocal images. Images were acquired with a resolution of 1024 pixels × 1024 pixels and a pixel-to-μm ratio of 1 pixel = 0.21 μm. Confocal slices were set between 0.17 (mainly for 3D reconstruction, Supplementary Fig. 3a) to 0.5 μm apart. The cell volume can then be approximated as

10 $$V=\int _{0}^{H}A\left(z\right){{\rm {d}}z}\cong \mathop{\sum} \limits_{z}A(z)\,\delta z$$

in which *A* is the measured cross-sectional area for each confocal slice; *H* is the cell height; $\delta z$ is the height difference between each confocal slice (0.5 μm). *A(z)* is quantified via the traced fluorescent signals from the cell membrane dye using MATLAB image analysis package, similar to the method described above under the subsection “Quantitative image analysis”.

3D reconstruction (Supplementary Fig. 3a) was done by triangulating surfaces based on confocal slices using MATLAB packages.

### RNA sequencing and analysis

Total RNA was extracted by adding 0.3 mL of TRIzol (Ambion) directly to the cell cultures (for 2d-U, 5d-U and 2 + 3d-C cells), immediately after removing the culture media (and the compression device for 2 + 3d-C cells). F cells were directly collected into 0.3 mL of TRIzol during sorting. The samples were then purified using Direct-zol RNA Microprep kit (Zymo research), reverse-transcribed to cDNA and amplified using SMART-seq v4 Ultra Low Input RNA kit (Takarabio). Amplified cDNA was used to prepare libraries for sequencing at 75 base-pair single-end reads using the Illumina NextSeq 500 system. On average, 48 million reads per sample were obtained, with <20% of intergenic reads. We analyzed the data using nf-co RNA-seq v 1.4.2 bioinformatics pipeline, built in Nextflow (https://nf-co.re/rnaseq/1.4.2). This pipeline mapped the reads to the mouse mm10 genome, annotated each gene using GENCODE vM23, and totaled the exonic reads for each gene. The raw reads were normalized and compared (with respective *p-*value) via DESeq2.r package, which also computed the PCA scores for each sample. We conducted the pathway analysis through IPA (Qiagen), GO (DAVID), and Reactome using the upregulated genes in 2 + 3d-C cells (with *p* < 0.05*)*.

### Identification of overlapping upregulated genes in quiescent MuSCs

The “core” genes that characterize quiescent MuSCs feature are defined by finding the overlapping upregulated genes in nascent^29^ and steady-state^28^ RNA-seq results. The differentially upregulated genes in quiescent MuSCs for the respective work can be found in Table S1 of refs. 29 and 28. Then, the *p*-value for the overlapping upregulated genes between 2 + 3d-C cells (vs. 5d-U or F) and the identified “core” quiescent genes is calculated by Fisher’s exact method (MATLAB package, see subsection “Statistics and reproducibility”).

### Statistics and reproducibility

Unless otherwise addressed, all statistics were analyzed using MATLB packages. We utilized the student’s standard two-tail *t*-test (for two unpaired populations; MATLAB ttest2 function) or Kruskal–Wallis test (for multiple group comparisons; MATLAB kruskalwallis and multcompare functions) to evaluate the significance of the differences between populations where each cell or field of view was considered as a single data point. The *t*-test was used to evaluate the *p* values for Fig. 1k; Supplementary Fig. 5a, where a single field of view was considered one data point; and Figs. 2b–f, g, h, j, k and 3g, h; Supplementary Figs. 1b–e, 3b, c and 6c, i where a single cell was considered a data point. The Kruskal–Wallis test was used to evaluate the *p*-value for Fig. 1e, where a single field of view was considered a data point; and Fig. 4c, e; Supplementary Fig. 4i, where a single cell was considered a data point. To assess the significance of the difference between populational fractions, we used two-tail Cochran–Mantel–Haenzel tests^54^, where we considered the total populational fractions over all the experimental repeats (Figs. 1c, g, i, l, 2e, i, 4b, d; Supplementary Figs. 4eand 6d, g). Fisher’s exact test was used to determine the significance of the gene overlap (Fig. 3d, Supplementary Fig. 7e), as well as pathway enrichment (which is calculated by each respective pipeline, Fig. 3e, Supplementary Fig. 7c, d, f). The significance of differentially expressed genes was calculated by DEseq2.r package (by assuming a negative binomial model).

### Reporting summary

Further information on research design is available in the Nature Portfolio Reporting Summary linked to this article.

## Supplementary information

Supplementary Information

Description of Additional Supplementary Files

Supplementary Data

Reporting Summary

## Data Availability

Source data for the figure panels (main figure and supplementary figure) are provided as Supplementary Data.xlsx (with corresponding panels labeled in the sheet names). Raw RNA sequencing data have been submitted to the NCBI Gene Expression Omnibus (GEO) with accession number GSE196101. The comparison of normalized reads in terms of Log2 difference and corresponding *p-*value is also included in the Supplementary Data.xlsx, under the sheet name: “Fig. 3 Gene_Log2FoldChange” (generated by DESeq2.r package; see Code Availability). The normalized gene reads for quiescent MuSCs, activated MuSCs and Notch downstream genes are labeled as Supplementary Tables 1–3, respectively (see Supplementary Information). The raw strain–stress data obtained from AFM experiment for cell tension estimation is available on GitHub (https://github.com/taojiaxiang1991/Code_Sharing_AFM). All other data are available from the corresponding author (or other sources, as applicable) on reasonable request.

## Code availability

We use MATLAB R2021b for all the analyses unless otherwise addressed. RNA-seq data were processed using nf-co RNA-seq v 1.4.2 bioinformatics pipeline, built in Nextflow (https://nf-co.re/rnaseq/1.4.2; see RNA Sequencing and Analysis). We run DESeq2.r package in R 4.04 version. Sample codes for epifluorescence boundary tracing (to identify positive signals for Pax7 and MyoD), bright field cell boundary tracing (mainly used to quantify nuclear vs. cytoplasmic Notch ratio), and cellular tension estimation from AFM strain–stress data are available on GitHub. (Epifluorescence boundary tracing and positive Pax7 and MyoD signals identification, together with sample sets: https://github.com/taojiaxiang1991/Fluorescent-boundary-tracing-and-cellular-fate-determination--Pax7-and-MyoD--, bright field cell boundary tracing to quantify Notch signals together with sample sets: https://github.com/taojiaxiang1991/DIC_boundary_tracing_for_Notch, cell tension estimation from the strain-stress data of AFM: https://github.com/taojiaxiang1991/Code_Sharing_AFM).
