# Supplementary material for: Mechanical compression creates a quiescent muscle stem cell niche
Source: Commun Biol. 2023 Jan 13;6:43. doi: 10.1038/s42003-023-04411-2 (PMC9839757; doi:10.1038/s42003-023-04411-2)
Supplement: Supplementary file 1 — Supplementary Information [file 42003_2023_4411_MOESM1_ESM.pdf]

Supplementary Information:

Supplementary Figures 1-10 and Supplementary Tables 1-3

Mechanical Compression Creates a Quiescent Muscle Stem Cell Niche

Authors: [1]Jiaxiang Tao, [2,3]Mohammad Ikbal Choudhury*, [2,3]Debonil Maity*, [4]Taeki Kim, [2,3,5]Sean X. Sun and [1,6]Chen-Ming Fan

Affiliations:

1. Embryology Department, Carnegie Institution for Science, 3520 San Martin Drive, Baltimore, MD 21218, USA.
2. Department of Mechanical Engineering, Johns Hopkins University, Baltimore, MD, 21218, USA
3. Institution for NanoBioTechnology, Johns Hopkins University, Baltimore, MD, 21218, USA
4. Department of Civil & Systems Engineering, Johns Hopkins University, Baltimore, MD, 21218, USA
5. Center for Cell Dynamics (CCD), Johns Hopkins School of Medicine, Baltimore, MD, 21205, USA
6. Department of Biology, Johns Hopkins University, Baltimore, MD 21218, USA.

* These authors contributed equally.

Corresponding author: Chen-Ming Fan; fan@carnegiescience.edu

Supplementary Figure 1: Cell dimension measurement for *in vivo* intravital imaging of Pax7-YFP cells (a), in both cell height (b) and axial length (c).

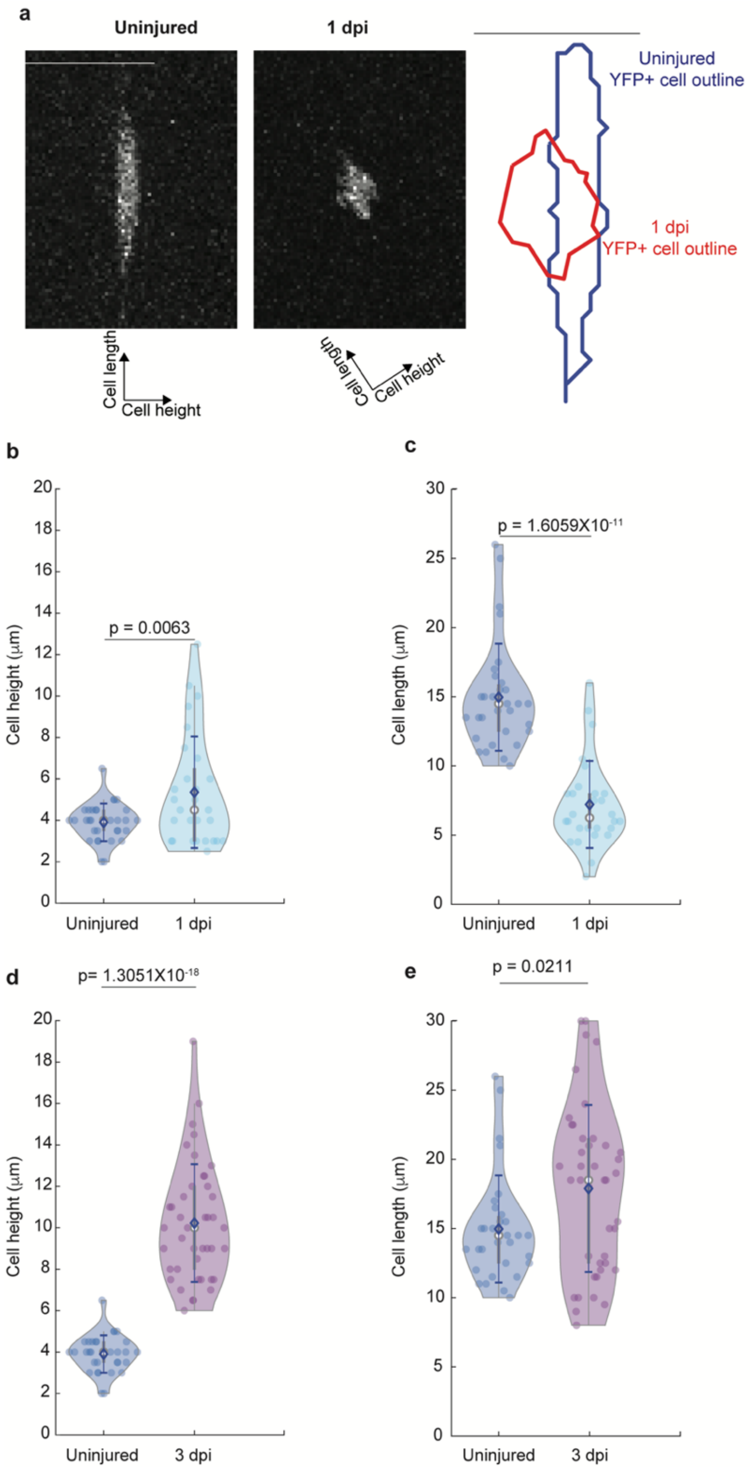

a, Representative images of uninjured and 1 dpi cells (Left panel, scale bar, 20 μm, with dimensional direction labeled) and their corresponding shape comparison (Right panel, scale bar, 10 μm). **b-e,** Quantitative measurements of YFP^+ cells (uninjured, 1 dpi, and 3 dpi) in terms of cell height **(b,d)** and cell length **(c,e)**: 31 cells from uninjured muscle: 28 cells from 1 dpi muscle and 42 cells from 3 dpi. Data is presented with mean $\pm$s.d. p-value was assessed with student's two-tail t-test using MATLAB. Comparison was considered significant if $p \leq 0.05$. This data was from Ref. 6. We used it with permission.

Supplementary Figure 2: Illustration and dimensions of the compression device.

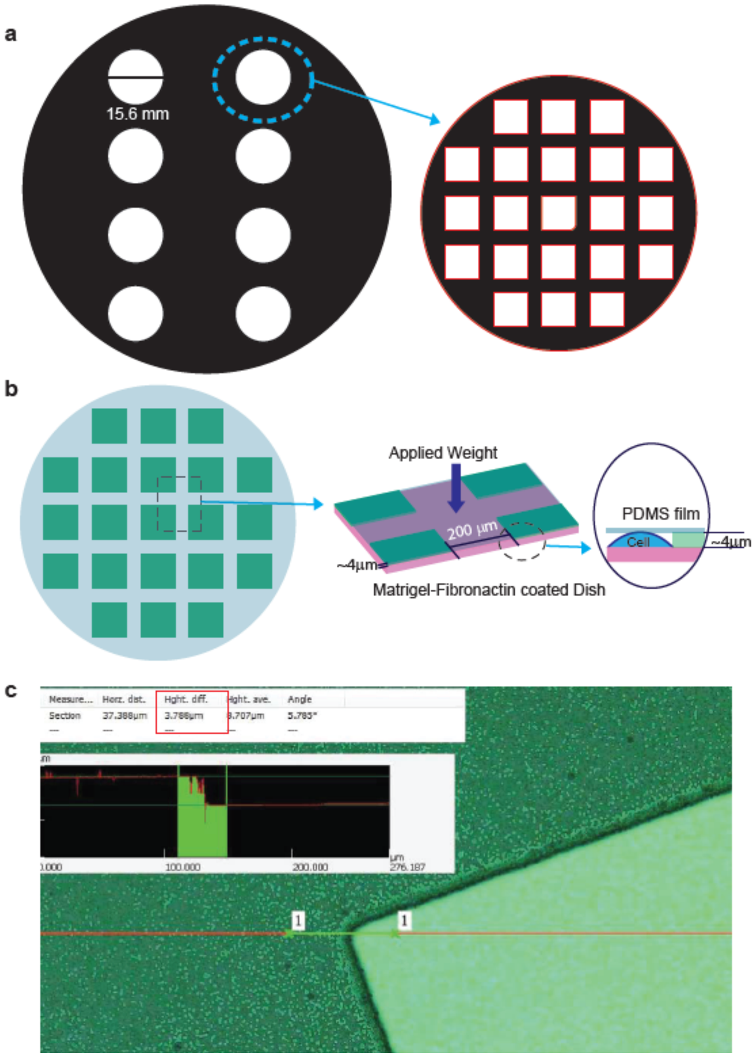

a-b, Illustration of compression device mold on a silicon wafer **(a),** and designed pillar pattern **(b)**. **c,** Top view of compression pillar. **c,** Height measurement of one pillar on the silicon wafer.

Supplementary Figure 3: 3D confocal measurement of cell height and volume.

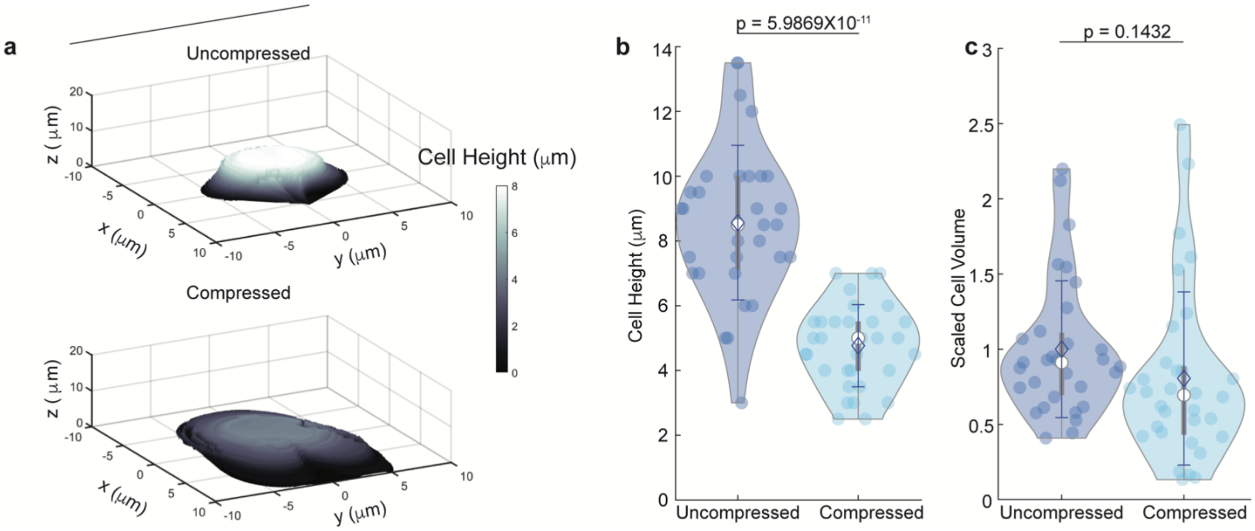

a, 3D rendering of uncompressed and compressed cell shape based on confocal data (Scale bar, 20 μm. This scale bar is the projection on the x-y plane)**. b-c,** Measured cell height **(b)** and volume **(c)** for uncompressed and compressed cells. (n = 5 for both uncompressed and compressed cells. 30 cells for uncompressed and 33 cells for compressed). Data is presented with mean $\pm$s.d. *p*-value was assessed with the student's two-tail t-test using MATLAB. The comparison was considered significant if $p \leq 0.05$. Cell volume is scaled with the mean volume of the uncompressed cells.

Supplementary Figure 4: Split-channel representative images of MuSCs and others.

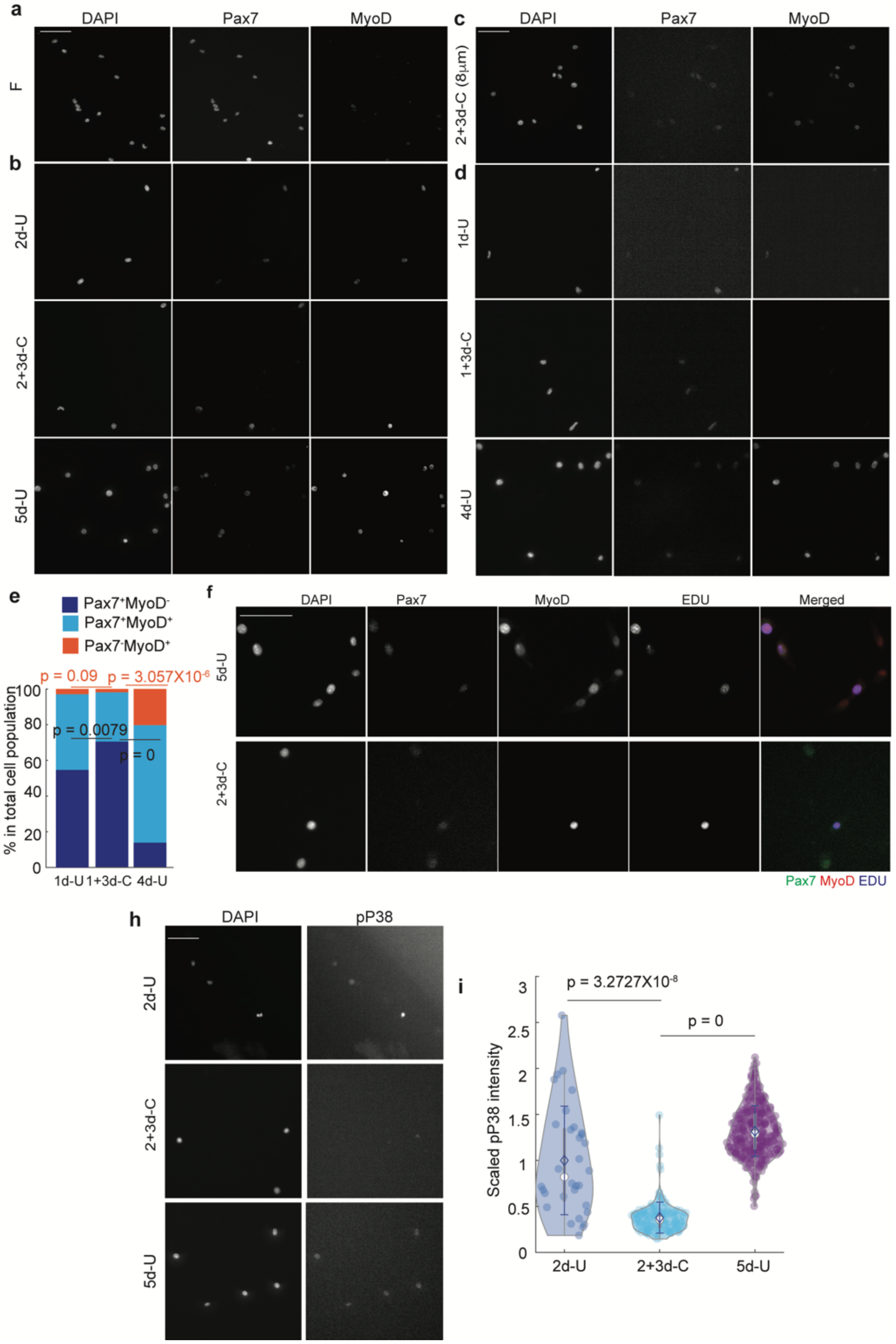

a-d , Representative DAPI, Pax7, and MyoD channels for F (Cytospinned) **(a)**, 2d-U, 2+3d-C, 5d-U **(b)**, 2+3d-C (8μm) **(c)**, 1d-U, 1+3d-C, and 4d-U **(d)** cells (Images for 5d-U and 2+3d-C are the split channel images for **Fig. 1b.** Scale bar, 25 μm). **e,** Cell fate evaluation of 1d-U, 4d-U, and 1+3d-C cells. (n = 3 for 1d-U, of total 87 cells; n = 3 for 1+3d-C, of total 105 cells; n = 3 for 4d-U, 4,430 cells). **f,** Representative images of DAPI, Pax7, MyoD, and EDU for 5d-U and 2+3d-C (same example as in **Fig. 1f.** Scale bar, 25 μm). **g,h,** Representative images **(g)** for pP38 staining of 2d-U, 5d-U, and 2+3d-C cells and the respective intensity quantification (Scale bar, 25 μm) **(h)**. n = 3 for both 2d-U and 5d-U cells; of total 32 cells for 2d-U and 315 cells for 5d-U. n = 6 for 2+3d-C cells; of total 168 cells. Data in **(e)** was presented with an overall fraction counting every cell from all experimental repeats. p-value was assessed by a two-tailed Cochran-Mantel-Haenszel test using MATLAB. Data in **(h)** was scaled by the mean pP38 intensity of the 2d-U cells. p-value was assessed by the Kruskal-Wallis test using MATLAB. The comparison was considered significant if $p \leq 0.05$.

Supplementary Figure 5: Additional representative images for the post-compressed, 7d-U and 7+d-C cells.

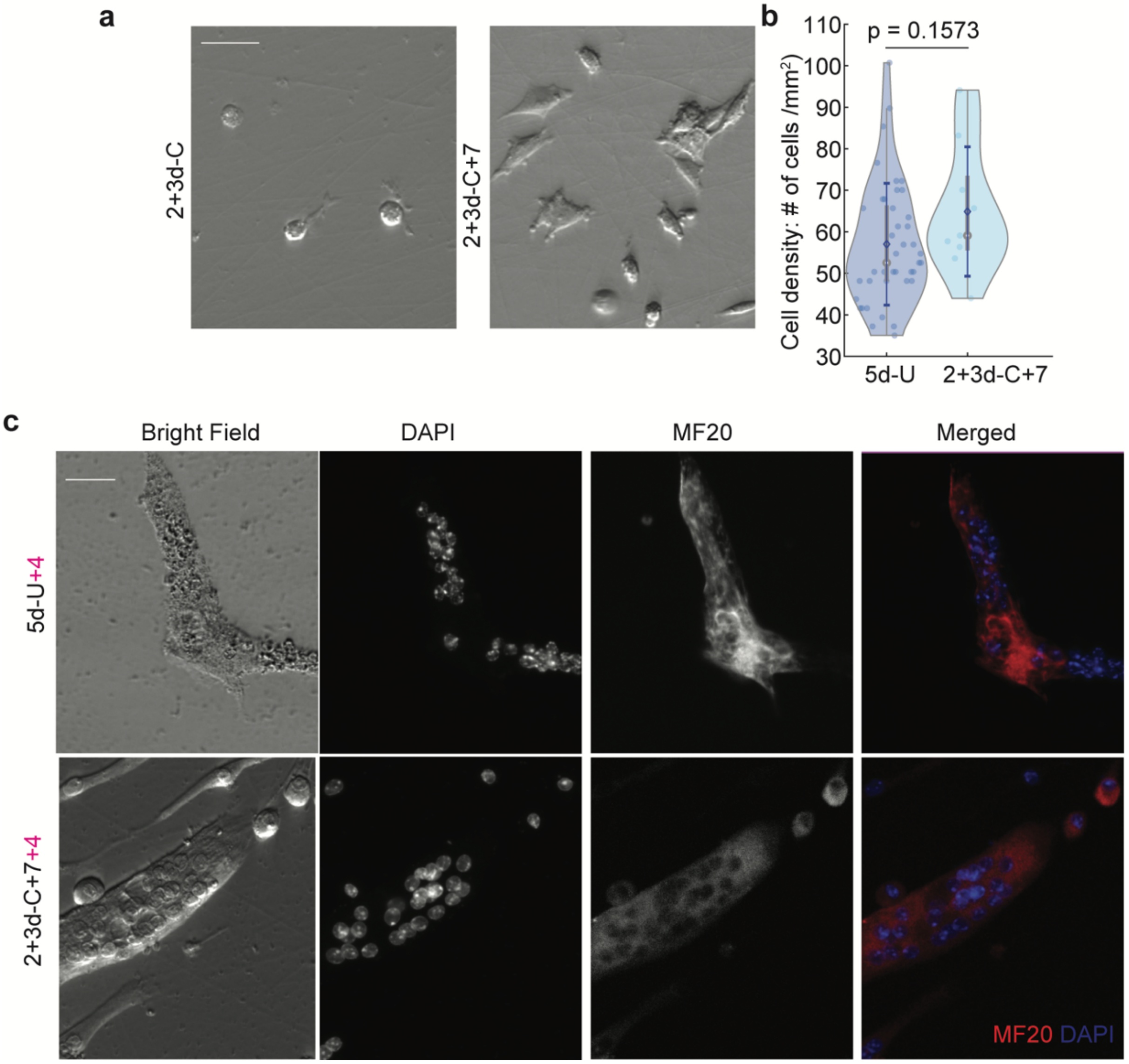

a, Bright-field example of cells right after removing compression pillar (2+3d-C) and after 7-day culturing after compression device's removal (2+3d-C+7) (Scale bar, 25 μm). **b,** Cell density comparison between 5d-U and 2+3d-C+7. **c,** Examples 5d-U and 2+3d-C+7 cells after 4-day culturing in differentiation medium, designated as 5d-U+4 and 2+3d-C+7+4, respectively (Scale bar, 25 μm). **d,** Representative images (split-channel images and merged-channel images) of

Pax7 and MyoD staining for 7d-U and 7+3d-C cells (Scale bar, 25 μm). **(b)** The sample selected for cell density quantification is the same sample presented in **Fig. 1j,k.** Data is presented with $\pm$s.d. Ten fields of view were selected for 2+3d-C+7+4 sample and Forty-two fields of view were selected for 5d-U+4 samples. p-value was assessed with student's two-tail t-test using MATLAB. Comparison was considered significant if $p \leq 0.05$

Supplementary Figure 6: Model illustration and additional analysis and repersentaitve images for tension manipulation experiments on MuSCs.

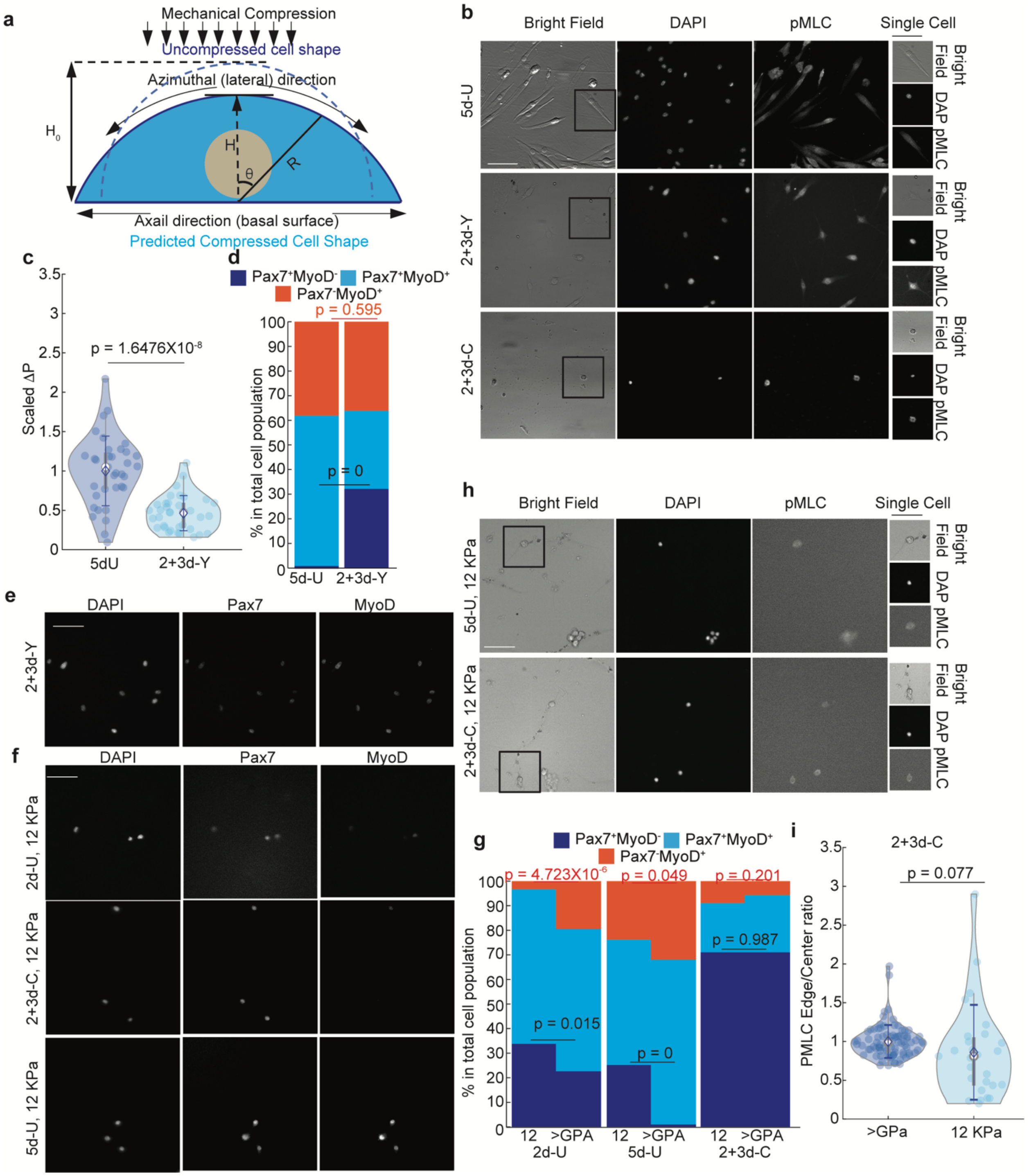

a, Cell surface is discretized by azimuthal radius, *R* and azimuthal angle, θ. This illustration shows uncompressed cell shape (dotted line, with height H_0), and predicted cell shape with a height, *H*. **b,** Representative images of pMLC distribution for 5d-U, 2+3d-C and 2+3d-Y cells seeded on plastic (Scale bar, 25 μm). **c,** Comparison of measured pressure between 5d-U and 2+3d-Y cells by AFM. (5d-U cells pressure data is the same as the plastic data in **Fig. 2g.** n= 7 for 2+3d-Y sets, of total 35 cells). **d,** Cell fate comparison between 5d-U and 2+3d-Y cells. (2+3d-Y's cell fate data is the same as in **Fig. 2e;** n = 3 for 5d-U sets, of total 985 cells). **e,f,** Representative images of Pax7 and MyoD expression for 2+3d-Y cells **(e)** and 2d-U, 5d-U, and 2+3d-C cells seeded on 12 KPa hydrogel **(f)** (Scale bar, 25 μm). **g,** Cell fate comparisons of 2d-U, 5d-U and 2+3d-C between cells seeded on plastic and 12 KPa hydrogel. (All plastic-seeded cell data is the same as in **Fig. 1c**. All 12 KPa hydrogel-seeded cell data is the same as in **Fig. 2i**). **h,** Representative images of pMLC distribution for 5d-U and 2+3d-C cells seeded on 12KPa hydrogel (Scale bar, 25 μm). **i,** scaled pMLC Edge/Center ratio for 2+3d-C cells seeded on plastic (>GPa) and 12 KPa hydrogel. (Same data presented in **Fig. 2c** for cells seeded on plastic and **Fig. 2k** for cells seeded on 12 KPa hydrogel.) Data in **(c)** and **(i)** is presented with ±s.d. *p*-value was assessed with student's two-tail t-test using MATLAB. Data in **(d)** and **(g)** is presented by overall fraction. *p*-value was assessed based on two-tailed Cochran-Mantel-Haenszel test using MATLAB. Comparison was considered significant if $p \leq 0.05$

Supplementary Figure 7: Additional RNA-seq analysis.

a

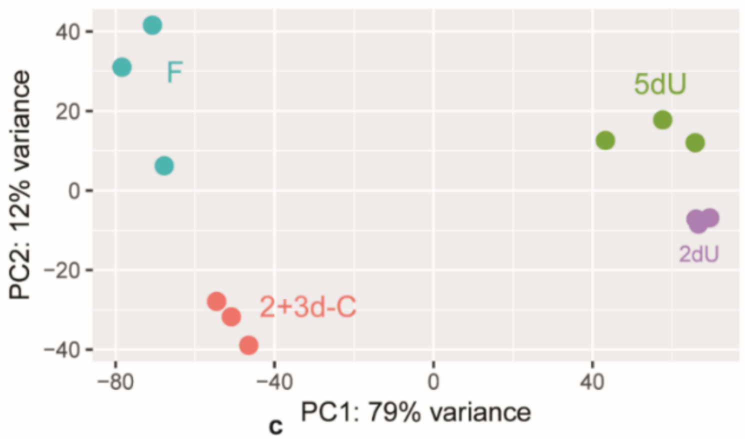

b

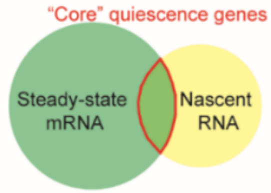

c

Pathway	p value	
	IPA	Reactom
G-protein	GP6: 0.0024	CAM-PDE1: 0.0134
Rho	0.025	0.045
Notch	0.0076	0.0019<br>Notch3: 0.0121<br>Notch4: 0.0027<br>NICD1: 0.0086<br>NICD3: 0.00125<br>NICD4: 0.0027
Hedgehog	Sonic: 0.038	0.0121
ECM-related	X	Organization: $3.8X10^{-4}$<br>Proteoglycans:0.03
Integrin	X	0.041

d

Pathway	p values: GO (DAVID)		p values: IPA	
	Compare to 5d-U	Compare to 2d-U	Compare to 5d-U	Compare to 2d-U
G-protein	cAMP: $5.75X10^{-6}$	cAMP: $1.8X10^{-5}$<br>GPCR: 0.011	GPCR: 0.002<br>cAMP: 0.0021	GPCR: $6.31X10^{-10}$<br>GP6: $1.45X10^{-10}$<br>cAMP: $1.15X10^{-9}$
Cell mechanics	Mechanical stimulus: 1.48X10-4<br>Rho: 0.0011<br>Calcium: 0.036<br>Actin cytoskeleton: 0.0195	Mechanical stimulus: $1.66X10^{-6}$<br>Cell shape: $1.92X10^{-6}$<br>Actin cytoskeleton: 0.0073<br>Osmotic stress: $8.9X10^{-5}$<br>Rho: $2.96X10^{-4}$	Rho: $8.51X10^{-9}$<br>Calcium transport: 0.019<br>RAC: $6.03X10^{-4}$<br>RHOA: 0.0074<br>Actin cytoskeleton: $4.47X10^{-5}$	Rho: $1.95X10^{-9}$<br>RAC: $2.24X10^{-6}$<br>RHOA: $2.95X10^{-6}$<br>Actin cytoskeleton: $1.95X10^{-9}$
WNT	WNT: 0.0032<br>Canonical: 0.0012	WNT: 0.019<br>Canonical: $2.07X10^{-4}$	Ca+: $6.92X10^{-6}$	Ca+: $6.03X10^{-7}$
Notch	$1.09X10^{-4}$	$9.23X10^{-6}$	$9.12X10^{-5}$	$1.25X10^{-5}$
Growth factor	TGF-beta: $1.23X10^{-4}$	TGF-beta: $7.22X10^{-9}$	FGF: 0.0012<br>PDGF: 0.0012	NGF: $1.35X10^{-7}$<br>HGF: $5.01X10^{-7}$<br>TGF-beta: $1.2X10^{-5}$<br>FGF: $1.82X10^{-6}$
Integrin	X	0.024	$2.34X10^{-6}$<br>ILK: $1.58X10^{-4}$	$5.37X10^{-7}$

e

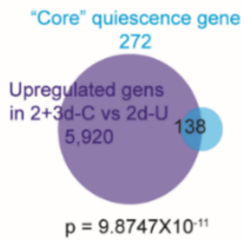

f

Pathway	p value	
	IPA	Reactom
G-protein	GP-6: $7.14X10^{-4}$<br>GPCR: 0.0437	Cam-PDE1: 0.00381<br>G-alpha: 0.0464
Actin-related	Nucleation: 0.0155<br>Rho: 0.0275	X
Notch signaling	0.0191	NICD4: $2.73X10^{-4}$<br>NICD1: $5.14X10^{-4}$<br>Notch3: $8.03X10^{-4}$<br>Notch1: 0.0028<br>Notch4: 0.00411<br>$1.94X10^{-4}$
Integrin	0.0316	0.00381
ECM-related	X	Degradation: 0.007<br>Proteoglycans: 0.002

a, PCA plot for 5d-U, F and 2+3d-C samples. **b,** Comparison of upregulated genes between steady-state mRNA and nascent mRNA, respectively, based on Ref. 28 and 29, respectively. **c,** Pathway analyses on "core" quiescence genes. **d,** Pathway analyses on all upregulated genes in 2+3d-C compared to 5d-U or F and 2d-U cells. **e,** Comparison between upregulated genes in 2+3d-C cells, relative to 2d-U cells, and "core" quiescence genes. **f,** Pathway analysis on the overlapped "core" quiescence genes in **(e)**. *p*-values in **(c)**, **(d)**, and **(f)** were calculated from each respective pipeline. The pathway is significantly enriched if $p<0.05$; otherwise, a "X" is marked. *p*-value in **(e)** was calculated by Fisher's extract method.

Supplementary Figure 8: Representative images related to Notch pathway investigation.

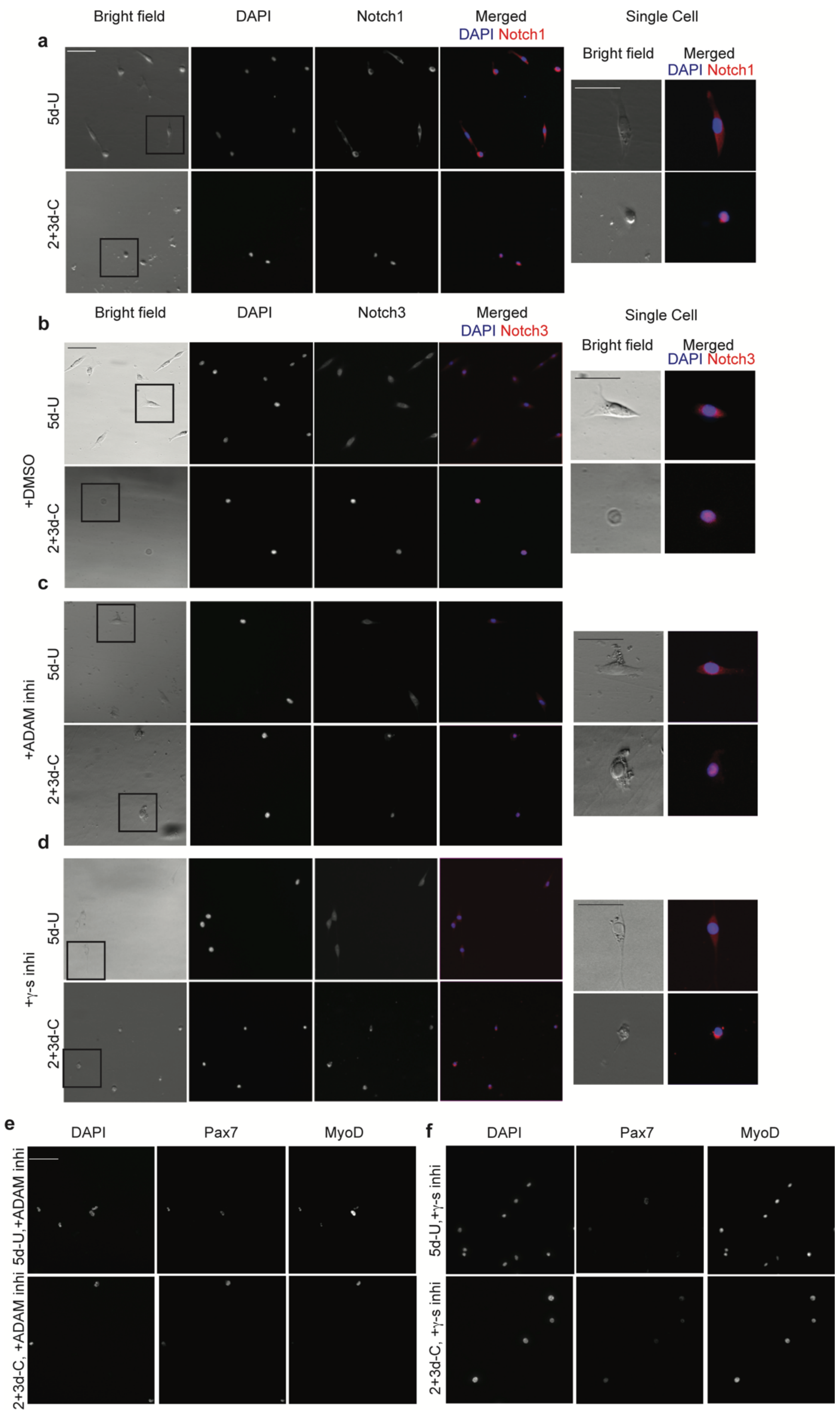

a,b, Representative images of Notch1 **(a)** and Notch3 **(b)** of 5d-U and 2+3d-C cells (Scale bar, 25 μm). **c,d,** Representative images of Notch3 in 5d-U and 2+3d-C cells when treated with ADAM inhi **(c)** and γ-s inhi **(d).** (Scale bar, 25 μm). **e,f,** Representative images of Pax7 and MyoD for 5d-U and 2+3d-C cells when treated with ADAM inhi **(e)** and γ-s inhi **(f).** (Scale bar, 25 μm).

Supplementary Figure 9: Technical details of AFM data-fitting.

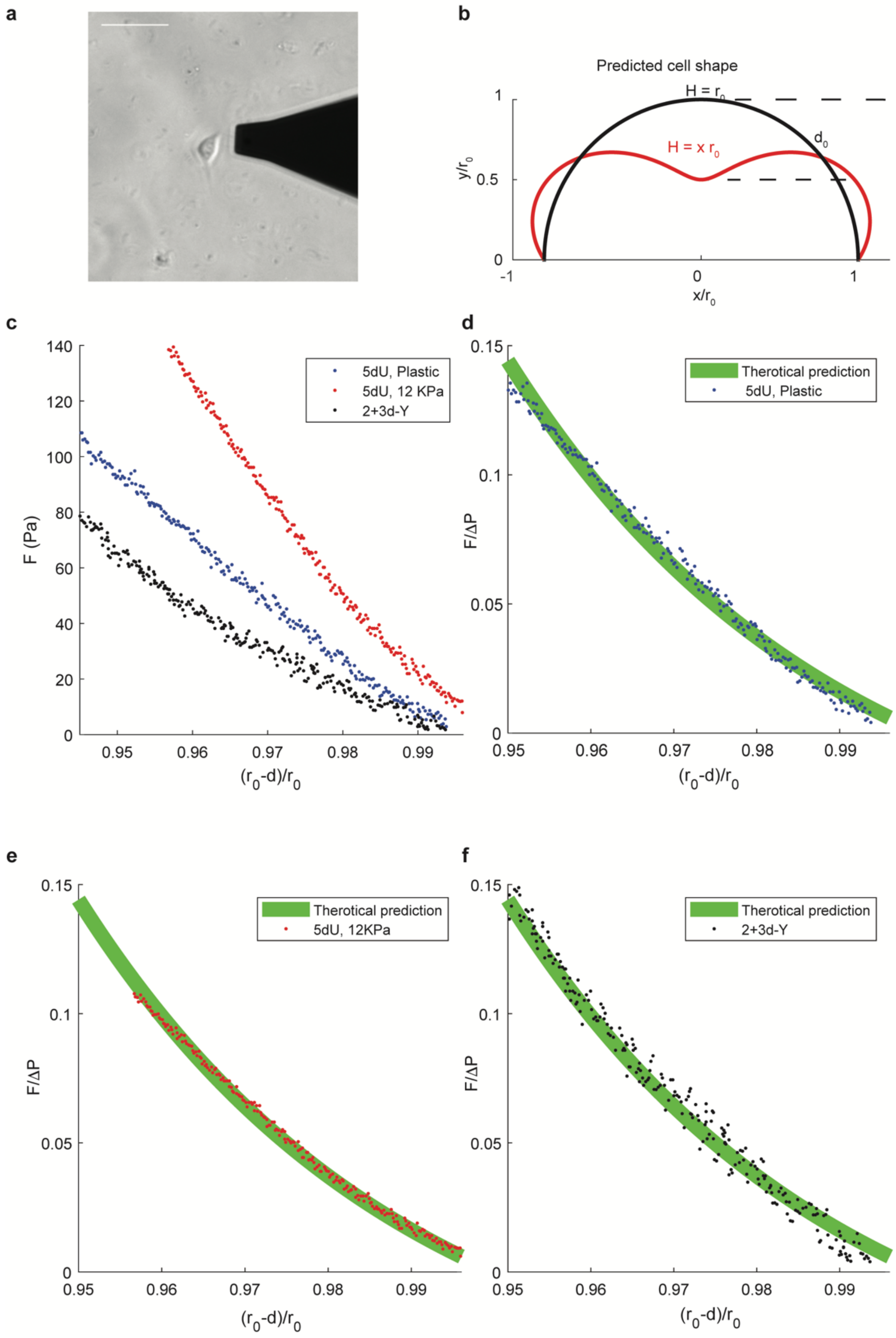

a, Illustration of AFM cantilever approaching a cell (Scale bar, 20 μm). **b,** Theoretical cell shape before (black) and after (red line) indentation. **c,** Example of indentation strain-responding stress (F) curves of 5d-U cells on plastic and 12 KPa hydrogel, and 2+3d-Y cells. **d-f,** Fitting indentation curves for 5d-U cells on plastic **(d),** and on 12 KPa hydrogel **(e)** and 2+3d-Y cells **(f)** with theoretical indentation strain-$\frac{F}{\Delta P}$ curve to calculate ΔP.

Supplementary Figure 10: Cell boundary tracing and quantification for immunostaining images.

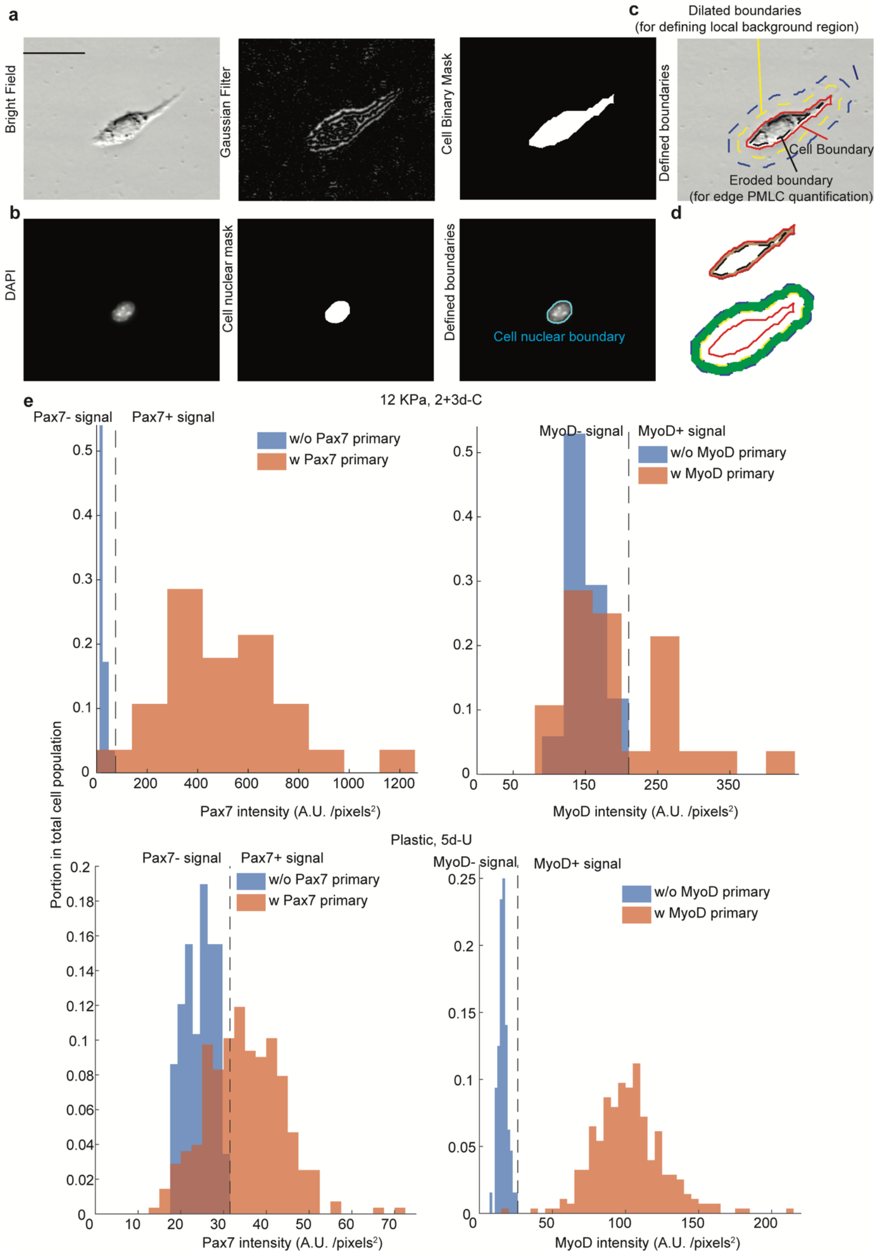

a, Example of cell-boundary tracing using bright field. **b,** Example of cell nucleus boundary tracing using DAPI channel. **c-d,** traced cell boundary shown in **(a)** and the respective dilated/eroded boundary. **e,** Example of Pax7 and MyoD intensity histograms for 2+3d-C cells on 12 KPa hydrogel and 5d-U cells on plastic.

Supplementary Table 1: Normalized reads (RPKM) for MuSCs quiescent genes (Fig. 3b)

Gene Name	5d-U, 1	5d-U,2	5d-U, 3	2d-U,1	2d-U,2	2d-U,3	2+3d-C,1	2+3d-C,2	2+3d-C,3	F,1	F,2	F,3
**Pax7**	845.1699	312.988	702.0297	970.3942	847.9054	920.4957	2190.795	2263.341	1650.58	439.3624	606.6024	284.5239
**Calcr**	30.57462	0	3.358994	3.868039	0	0	2038.514	736.9016	1228.917	475.2287	2335.244	110.5345
**Tenm4**	61.14924	6.853753	50.38491	17.40617	0	1.834875	2411.674	1706.02	3918.106	732.8141	669.7171	390.9645
**Col5a1**	330.8611	316.0342	304.5488	385.3534	164.2539	492.3582	5623.186	1362.339	6009.007	6556.2	1822.145	2858.544
**Col5a3**	46.95388	11.42292	23.51296	38.68039	72.13855	0	6144.734	860.7506	4992.787	7478.128	1144.246	3027.416
**Col6a1**	60.05729	13.70751	16.79497	8.703087	0	0	5437.335	944.3487	6047.566	30905.35	4578.153	26549.77
**Col6a2**	31.66657	3.046112	13.43598	76.87727	49.94207	0	1700.87	380.8357	1751.331	19175.44	1356.966	13071.72
**Cdkn1c**	29.48267	109.66	304.5488	10.63711	0	0	664.0983	2724.678	1497.587	1521.873	1935.517	1998.832
**Gas1**	395.2862	1484.98	971.8689	983.9323	1201.939	888.0795	3921.342	2117.818	1665.506	3624.129	3508.71	4619.931
**Pmp22**	599.481	164.4901	222.8133	450.143	549.3628	558.4137	7293.405	14208.58	4046.222	17964.95	21605.1	20312.76
**Ptprz1**	200.9189	0	24.63262	51.73502	27.7456	0	2018.567	709.0356	1052.291	735.2595	1313.721	369.4717
**Tek**	63.33314	12.18445	16.79497	33.36183	0	25.68825	1730.548	191.966	1733.918	4003.985	3666.497	2756.197
**Apoe**	358.1598	28.93807	208.2576	166.8092	172.0227	129.0529	33008.85	102200.2	62898.67	16513.99	35339.56	17691.66
**Rgs2**	193.2753	28.17654	139.9581	33.84534	37.73401	47.70675	500.1414	1142.507	426.6382	1229.237	4752.303	823.8912
**Spry1**	571.0902	335.0724	278.7965	1416.186	1464.967	1237.317	23414.21	20648.73	17092.89	6645.866	9478.893	5319.983

Supplementary Table 2: Normalized reads (RPKM) for MuSCs quiescent genes (Fig. 3c)

Gene Name	5d-U, 1	5d-U,2	5d-U, 3	2d-U,1	2d-U,2	2d-U,3	2+3d-C,1	2+3d-C,2	2+3d-C,3	F,1	F,2	F,3
**Myog**	14.19536	19131.11	37916.32	83.16283	19.97683	73.395	0.486519	0	0	161.3984	37.4013	10988.97
**Mki67**	1131.261	683.8522	957.3133	498.01	752.4606	601.839	0.973038	0	0	245.3582	148.4364	227.2097
**Mcm2**	3490.967	6211.785	1538.419	11564.95	11977.22	12115.07	178.0659	74.3094	0	411.6475	425.4398	720.521
**Bub1**	1722.006	1796.445	972.9886	1787.517	2431.624	1923.561	0	0	0	71.73264	46.75163	98.25286
**Plk1**	3414.53	4740.512	3204.48	6553.908	5688.957	6173.743	0.486519	0	0	44.8329	43.24526	355.1432
**Ccne1**	1751.489	3506.075	3342.199	3896.082	4000.915	3609.199	41.84063	0	2.487687	100.2627	112.2039	373.5656
**Ccnb1**	5960.959	9004.308	6627.295	8560.453	9507.861	9334.01	33.56981	3.096225	0	92.11123	156.618	567.0009
**Gmnn**	1668.501	2100.294	5712.529	1782.199	1908.897	1919.891	0	0	99.50746	54.61462	99.34721	192.4119
**Cdc6**	2283.269	1690.592	1301.05	4009.706	3731.228	4310.733	0	0	97.01978	185.0376	330.7678	283.5004
**Cdc45**	2032.12	2366.068	1926.943	3110.387	2826.721	2450.782	107.0342	182.6773	0	128.7927	188.1753	300.8994
**Cdc7**	911.7789	769.9049	357.173	1007.624	1059.882	974.9303	145.4692	151.715	24.87687	166.2893	246.6148	192.4119
**Top2a**	3622.001	4523.477	2950.316	4737.38	3576.962	3248.952	144.0096	77.40563	52.24142	371.7055	556.3444	839.2432

Supplementary Table 3: Normalized reads (RPKM) for Notch downstream genes (Fig. 3f)

Gene Name	5d-U, 1	5d-U,2	5d-U, 3	2d-U,1	2d-U,2	2d-U,3	2+3d-C,1	2+3d-C,2	2+3d-C,3	F,1	F,2	F,3
**Notch1**	187.8155	46.45321	69.41921	174.5452	39.95366	0	2667.097	229.1207	1467.735	3745.585	779.5834	1196.433
**Notch2**	376.723	78.43739	182.5053	25.62576	26.63577	132.111	2301.721	164.0999	1000.05	2208.224	473.3603	494.3347
**Notch3**	41.49413	41.88405	237.3689	42.54843	125.4101	114.9855	11886.14	2003.258	10218.17	2020.741	1514.753	733.8261
**Hey1**	38.21828	214.7509	301.1898	79.29479	106.5431	71.56013	1278.572	962.926	961.4908	1313.196	2262.779	1414.432
**Heyl**	24.02292	11.42292	59.34223	61.40511	19.97683	38.53238	10197.92	4576.221	22812.09	6838.24	6333.677	3651.731
**Hes6**	8910.318	45369.56	181827.9	10070.44	13580.91	9775.603	308.453	275.564	212.6972	146.7259	174.1498	8018.866
**App**	1523.271	2053.08	938.279	1085.952	690.3104	1154.136	6406.481	2204.512	7948.159	21391.81	11130.39	12500.63
**S1pr3**	34.94242	9.138337	8.957317	50.76801	44.39295	66.0555	14516.26	3371.789	12163.54	6355.675	10409.25	7468.241
**Dtx3**	561.2627	434.071	517.2851	612.6006	649.2469	588.9949	3819.173	5557.724	4495.25	1889.503	1008.666	2609.842
**Dtx4**	265.344	273.3886	136.5991	770.2232	402.8661	879.5168	3435.31	752.3827	899.2987	1103.704	1714.616	532.203
**Rcan2**	144.1375	70.82211	222.8133	117.4917	46.6126	130.8878	1428.906	1613.133	2191.652	1080.065	2709.257	1167.776
**Galnt11**	127.7582	278.7193	213.8559	243.6864	243.0514	351.0728	1799.147	1102.256	1422.957	661.0815	1018.017	756.3423
