# Supplementary material for: Mechanical compression creates a quiescent muscle stem cell niche
Source: Commun Biol. 2023 Jan 13;6:43. doi: 10.1038/s42003-023-04411-2 (PMC9839757; doi:10.1038/s42003-023-04411-2)
Supplement: Supplementary file 2 — Description of Additional Supplementary Files [file 42003_2023_4411_MOESM2_ESM.pdf]

Description of Additional Supplementary Files

File name: Supplementary Data 1
Description: Source data for the figure panels (main figures and supplementary figures).
