# Supplementary material for: Mechanical compression creates a quiescent muscle stem cell niche
Source: Commun Biol. 2023 Jan 13;6:43. doi: 10.1038/s42003-023-04411-2 (PMC9839757; doi:10.1038/s42003-023-04411-2)
Supplement: Supplementary file 4 — Reporting Summary [file 42003_2023_4411_MOESM4_ESM.pdf]

nature portfolio

Corresponding author(s): Chen-Ming Fan

Last updated by author(s): 11/07/2022

Reporting Summary

Nature Portfolio wishes to improve the reproducibility of the work that we publish. This form provides structure for consistency and transparency in reporting. For further information on Nature Portfolio policies, see our Editorial Policies and the Editorial Policy Checklist.

Statistics

For all statistical analyses, confirm that the following items are present in the figure legend, table legend, main text, or Methods section.

n/a	Confirmed	
☐	☒	The exact sample size (*n*) for each experimental group/condition, given as a discrete number and unit of measurement
☐	☒	A statement on whether measurements were taken from distinct samples or whether the same sample was measured repeatedly
☐	☒	The statistical test(s) used AND whether they are one- or two-sided<br>*Only common tests should be described solely by name; describe more complex techniques in the Methods section.*
☒	☐	A description of all covariates tested
☐	☒	A description of any assumptions or corrections, such as tests of normality and adjustment for multiple comparisons
☐	☒	A full description of the statistical parameters including central tendency (e.g. means) or other basic estimates (e.g. regression coefficient) AND variation (e.g. standard deviation) or associated estimates of uncertainty (e.g. confidence intervals)
☐	☒	For null hypothesis testing, the test statistic (e.g. *F*, *t*, *r*) with confidence intervals, effect sizes, degrees of freedom and *P* value noted<br>*Give P values as exact values whenever suitable.*
☒	☐	For Bayesian analysis, information on the choice of priors and Markov chain Monte Carlo settings
☒	☐	For hierarchical and complex designs, identification of the appropriate level for tests and full reporting of outcomes
☒	☐	Estimates of effect sizes (e.g. Cohen's *d*, Pearson's *r*), indicating how they were calculated

Our web collection on statistics for biologists contains articles on many of the points above.

Software and code

Policy information about availability of computer code

Data collection	NIS Element, ZEN, BD FACSDiva Software
Data analysis	MATLAB (2021b), DEseq2 (1.30.1) in R (4.0.4),. nf-co RNA-seq pipeline(1.4.2)

For manuscripts utilizing custom algorithms or software that are central to the research but not yet described in published literature, software must be made available to editors and reviewers. We strongly encourage code deposition in a community repository (e.g. GitHub). See the Nature Portfolio guidelines for submitting code & software for further information.

Data

Policy information about availability of data

All manuscripts must include a data availability statement. This statement should provide the following information, where applicable:

- Accession codes, unique identifiers, or web links for publicly available datasets
- A description of any restrictions on data availability
- For clinical datasets or third party data, please ensure that the statement adheres to our policy

Source data is provided with the submission. RNA sequencing data have been submitted to the NCBI Gene Expression Omnibus (GEO) with accession number GSE196101. All codes used for analyses are available upon reasonable request.

Human research participants

Policy information about studies involving human research participants and Sex and Gender in Research.

Reporting on sex and gender	N/A
Population characteristics	N/A
Recruitment	N/A
Ethics oversight	N/A

Note that full information on the approval of the study protocol must also be provided in the manuscript.

Field-specific reporting

Please select the one below that is the best fit for your research. If you are not sure, read the appropriate sections before making your selection.

☒ Life sciences ☐ Behavioural & social sciences ☐ Ecological, evolutionary & environmental sciences

For a reference copy of the document with all sections, see nature.com/documents/nr-reporting-summary-flat.pdf

Life sciences study design

All studies must disclose on these points even when the disclosure is negative.

Sample size	When populational means were compared, each cell or field of view was considered as a single data point (Fig. 1e,k; Extended Data Fig. 5a for the field of view; Fig. 2b,c,d,f,g,h,j,k; Fig. 3g,h; Fig. 4c,e; Extended Data Fig. 1b,c; Extended Data Fig 3.b,c; Extended Data Fig. 4i Extended Data Fig. 6c,i for single cell). When populational fractions were compared, we considered the total populational fractions over all the experimental repeats (Fig. 1c,g,i,l; Fig. 2e,i; Fig. 4b,d; Extended Data Fig. 4e; Extended Data Fig. 6d,g).
Data exclusions	No exclusions
Replication	Each set of experiment has been repeated at least three times. Additional repeats were done for low output experiments. All data collected from all the repeats were included.
Randomization	For each batch of sorted cells, they are randomly distributed for each experimental condition.
Blinding	N/A

Reporting for specific materials, systems and methods

We require information from authors about some types of materials, experimental systems and methods used in many studies. Here, indicate whether each material, system or method listed is relevant to your study. If you are not sure if a list item applies to your research, read the appropriate section before selecting a response.

Materials & experimental systems

n/a	Involved in the study	
☐	☒	Antibodies
☒	☐	Eukaryotic cell lines
☒	☐	Palaeontology and archaeology
☐	☒	Animals and other organisms
☒	☐	Clinical data
☒	☐	Dual use research of concern

Methods

n/a	Involved in the study	
☒	☐	ChIP-seq
☐	☒	Flow cytometry
☒	☐	MRI-based neuroimaging

Antibodies

Antibodies used	We used mouse IgG1 Pax7 primary antibody (Supernatant from hybridoma, DSHB). Primary antibody for MyoD was mouse IgG2b (Santa Cruz). Primary antibodies for Notch1 (Abcam), Notch3 (Abcam), and pMLC (Cell Signaling) were rabbit IgG. Primary antibody for pP38 (Cell Signaling) was rabbit IgG. Primary antibody for MF-20 was mouse IgG2b (Supernatant from hybridoma, DSHB).

Validation	It's validated by the company. Sources are provided in the supplementary material: Antibody Used section.

Animals and other research organisms

Policy information about studies involving animals; ARRIVE guidelines recommended for reporting animal research, and Sex and Gender in Research

Laboratory animals	Pax7-ZSGreen mouse, from Bosnakovski, et al., Stem Cells, 2008
Wild animals	N/A
Reporting on sex	Finding is not sex-based
Field-collected samples	N/A
Ethics oversight	All experimental procedures for the mouse were approved by Carnegie Institutional Animal Care and Use Committee (IACUC).

Note that full information on the approval of the study protocol must also be provided in the manuscript.

Flow Cytometry

Plots

Confirm that:

☒ The axis labels state the marker and fluorochrome used (e.g. CD4-FITC).

☒ The axis scales are clearly visible. Include numbers along axes only for bottom left plot of group (a 'group' is an analysis of identical markers).

☒ All plots are contour plots with outliers or pseudocolor plots.

☒ A numerical value for number of cells or percentage (with statistics) is provided.

Methodology

Sample preparation	Pax7+ cells are collected from the hindlimb muscle of Pax7-ZSGreen mice, through FACS sorting based on the protocol given by Liu, et al., Nat. Protoc 2015 (Ref. 51). Detailed description can be found in the method section of the manuscript
Instrument	FACSAria II (BD)
Software	BD FACSDiva Software
Cell population abundance	About 2% of the total muscle cells are Pax7+. Almost all collected cells are Pax7+. The purity of the sample was determined through cytospin, shown in Fig. 1a, main text.
Gating strategy	FACS gating strategy is shown in Fig. 1a, main text. Cell population was plotted in terms of FITC-A (Green) and a negative florescent channel in log10 scale (e. g., PE-A, Fig. 1a). The high FITC-A-expressing population (Green, Fig. 1a) away from the main line (i.e., autofluorescence line, Red, Fig. 1a) was ZSGreen+ population

☒ Tick this box to confirm that a figure exemplifying the gating strategy is provided in the Supplementary Information.
